# A novel p53 regulator, C16ORF72/TAPR1, buffers against telomerase inhibition

**DOI:** 10.1111/acel.13331

**Published:** 2021-03-04

**Authors:** Yahya Benslimane, María Sánchez‐Osuna, Jasmin Coulombe‐Huntington, Thierry Bertomeu, Danielle Henry, Caroline Huard, Éric Bonneil, Pierre Thibault, Mike Tyers, Lea Harrington

**Affiliations:** ^1^ Institute for Research in Immunology and Cancer Université de Montréal Montréal QC Canada; ^2^ Department of Chemistry Université de Montréal Montréal QC Canada; ^3^ Department of Medicine Université de Montréal Montréal QC Canada

**Keywords:** C16ORF72, CRISPR‐Cas9, genome‐wide screen, p53, synthetic‐sick‐lethal, telomerase inhibitor (BIBR1532), Telomere Attrition and P53 Response 1, telomere erosion

## Abstract

Telomere erosion in cells with insufficient levels of the telomerase reverse transcriptase (*TERT*), contributes to age‐associated tissue dysfunction and senescence, and p53 plays a crucial role in this response. We undertook a genome‐wide CRISPR screen to identify gene deletions that sensitized p53‐positive human cells to telomerase inhibition. We uncovered a previously unannotated gene, *C16ORF72*, which we term Telomere Attrition and p53 Response 1 (*TAPR1*), that exhibited a synthetic‐sick relationship with *TERT* loss. A subsequent genome‐wide CRISPR screen in *TAPR1*‐disrupted cells reciprocally identified *TERT* as a sensitizing gene deletion. Cells lacking *TAPR1* or *TERT* possessed elevated p53 levels and transcriptional signatures consistent with p53 upregulation. The elevated p53 response in *TERT*‐ or *TAPR1*‐deficient cells was exacerbated by treatment with the MDM2 inhibitor and p53 stabilizer nutlin‐3a and coincided with a further reduction in cell fitness. Importantly, the sensitivity to treatment with nutlin‐3a in *TERT*‐ or *TAPR1*‐deficient cells was rescued by loss of p53. These data suggest that TAPR1 buffers against the deleterious consequences of telomere erosion or DNA damage by constraining p53. These findings identify C16ORF72/TAPR1 as new regulator at the nexus of telomere integrity and p53 regulation.

## Graphical abstract

Telomere erosion leads to an upregulation of p53 that ultimately results in cell arrest or death. Via genome‐wide screens, we identified a previously unannotated gene, C16ORF72/TAPR1 (Telomere Attrition and P53 Response 1), that was synthetically sick with telomerase inhibition or TERT loss. TAPR1 deletion led to elevated p53 activation in response to DNA damage or telomere erosion, possibly via its interaction with the E3 ubiquitin ligase, HUWE1.

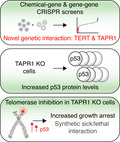

## 1 INTRODUCTION

Telomeres, which are the repetitive, G‐rich DNA sequences found at the ends of eukaryotic chromosomes, are a lynchpin of genome integrity. The loss of sufficient telomere reserves or end‐protection complexes elicits a DNA damage response that shares characteristics with the response to a double‐stranded DNA break (de Lange, 2018). In many organisms, this loss is prevented by the telomerase reverse transcriptase (TERT) and its associated telomerase RNA component (TERC), which add new telomeric DNA repeats to chromosome ends. The recruitment of telomerase to telomeres is highly regulated and depends on subunits of the telomere‐protective complex shelterin, POT1 and TPP1 (Aramburu et al., 2020). While *TERT* is expressed in stem/progenitor cells and in most cancers (Lorbeer & Hockemeyer, 2020), it is transcriptionally repressed in most adult human tissues (Roake & Artandi, 2020).

Primary human cells have a limited replicative capacity in culture, called cellular senescence or the Hayflick limit, which is tightly correlated with initial telomere length (Harley et al., 1994). When telomeres become sufficiently eroded, cells with functional p53 undergo induction of p21 and cell cycle arrest (Bunz et al., 1998; Herbig et al., 2004). Cells without functional p53 may temporarily avert senescence (called M1), but the eventual onset of telomere loss, fusions, or other genomic rearrangements lead to an M2 event called crisis (Harley et al., 1994). This crucial role of p53 in telomere‐induced or other types of senescence is context‐dependent and is influenced by mitochondrial activity, mTOR signaling and reactive oxygen species production (Maddocks & Vousden, 2011).

Genome‐wide screens in yeast have identified hundreds of genes that impact telomere length maintenance (Harari et al., 2020). Human gene networks that affect cellular senescence have also been identified in genome‐wide shRNA knockdown screens (Mazzucco et al., 2017; Wang et al., 2016). For example, the deubiquitinating enzyme USP28 is an important mediator that links p53 induction and the senescence‐associated secretory phenotype (Mazzucco et al., 2017) and plays a role in the response to DNA damage (Zhang et al., 2006). Despite these advances, the genetic landscape that helps human cells cope with eroded telomeres has remained elusive. Here, we exploited CRISPR‐Cas9 genome‐wide screens to identify genes that modulate cell fitness in the presence of critically eroded telomeres, which led to the discovery of a new modulator of the p53‐dependent response to telomere erosion, *C16ORF72*/*TAPR1*.

## 2 RESULTS

### 2.1 A genome‐wide CRISPR knockout screen identifies genetic dependencies of telomerase inhibition

**FIGURE 1:**
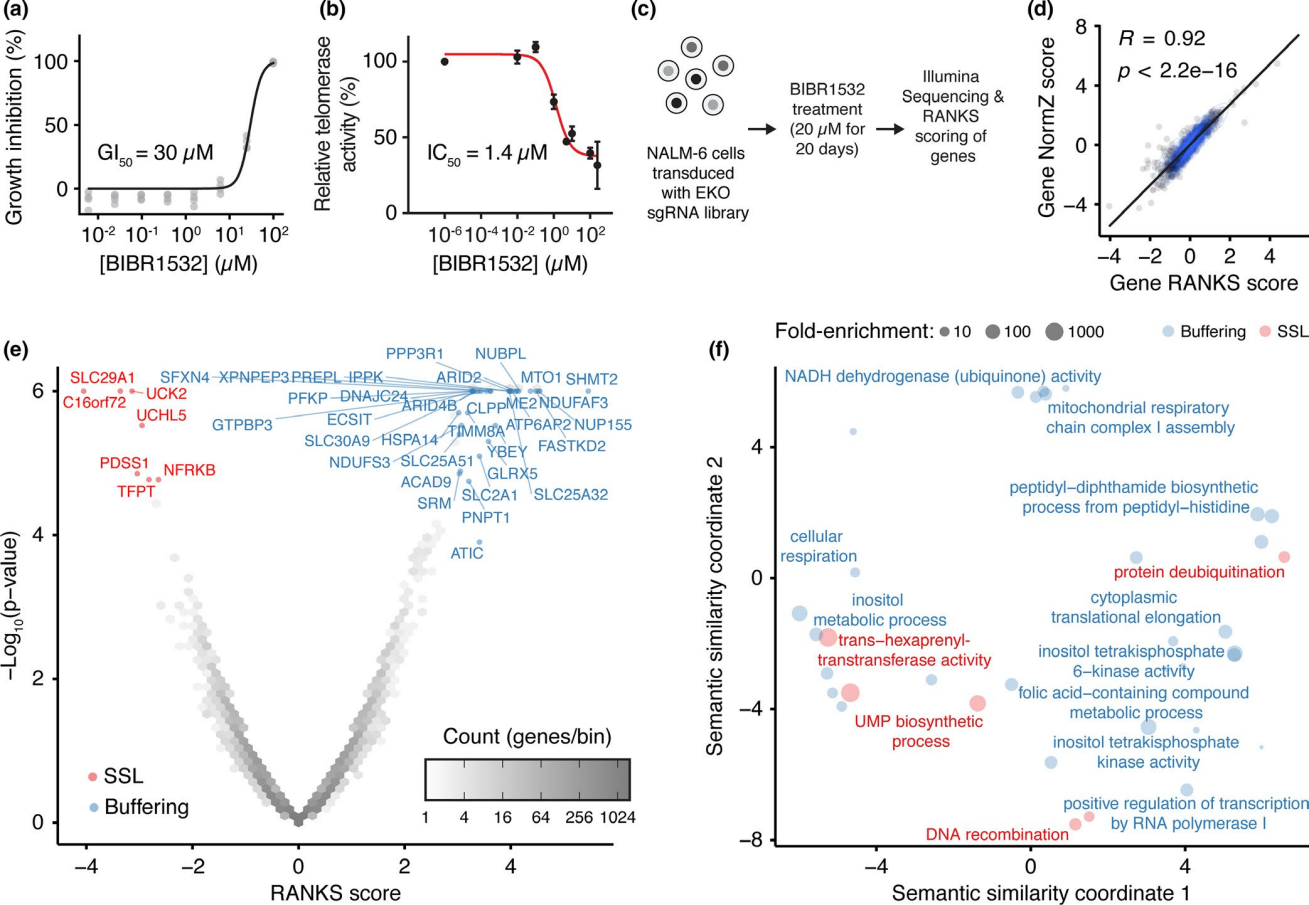
Genome‐wide CRISPR knockout screen identifies chemical‐genetic interactions with telomerase inhibition by BIBR1532. (a) Growth inhibition of NALM‐6 cells upon treatment with the indicated concentrations of BIBR1532 for 72 h (*n* = 4). (b) Inhibition of telomerase activity in NALM‐6 cell lysates by BIBR1532 measured by qTRAP (*n* = 3). (c) Genome‐wide CRISPR knockout screen schematic and genetic interaction identification using the RANKS algorithm. (d) Pearson correlation between the chemical‐genetic interaction for each gene with BIBR1532 (20 days, 20 µM) as analyzed by the RANKS or DrugZ algorithms. (e) Volcano plot showing the RANKS scores from each gene treated with BIBR1532 (20 µM) relative to the negative log‐transformed *p*‐value. Shades of gray in each hexagonal bin represent gene count and synthetic‐sick/lethal (SSL) chemical‐genetic interactions are labeled in red (RANKS < −2 & FDR < 0.05 for visualization purpose) while buffering interactions are labeled in blue (RANKS > 3 & FDR < 0.05 for visualization purpose). (f) Gene ontology (GO) term enrichment in the list of buffering (RANKS > 2 & FDR < 0.1, shown in blue) or SSL (RANKS < −2 & FDR < 0.1, shown in red) hits. The position of GO terms represents their semantic similarity and a subset is labeled to aid visualization

Given the known importance of p53 in the response to critically eroded telomeres, we chose to conduct a genome‐wide CRISPR screen in the NALM‐6 pre‐B ALL cell line because it possesses wild‐type p53 and is well suited to large‐scale genetic screens owing to the ability to grow in suspension and a near‐diploid karyotype (Bertomeu et al., 2017). We first established that deletion of *TERT* in NALM‐6 cells led to an eventual loss of proliferative capacity and onset of caspase activation concomitant with critical telomere erosion (Figure S1a‐c). We chose to inhibit telomerase with the small molecule BIBR1532 in a chemical‐genetic screen as it induces telomere erosion in numerous cell models and its specificity is well established, including a co‐crystal structure with *T. castaneum* TERT (Bryan et al., 2015). In NALM‐6 cells, we established the IC_50_ for telomerase inhibition by BIBR1532 in cell extracts was 1.4 µM, with a growth inhibition IC_50_ of 30 µM in culture, consistent with concentrations described in other studies (Figure 1a‐b; see also Appendix S1). To facilitate the identification of CRISPR‐induced indels that either exacerbated or buffered against telomerase inhibition, we chose a BIBR1532 concentration of 20 µM (~IC_30_) to conduct the genome‐wide screen. We confirmed this BIBR1532 concentration was sufficient to elicit telomere erosion after 20 days of treatment relative to the vehicle control, 0.1% DMSO (v/v; Figure S1c).

The genome‐wide screen was carried out using the previously described extended knockout (EKO) pooled CRISPR‐Cas9 sgRNA library transduced into an inducible Cas9 NALM‐6 cell clone (Benslimane et al., 2020; Bertomeu et al., 2017) treated with either 0.1% DMSO (v/v) or 20 µM BIBR1532 for 20 days (Figure 1c‐d). Several gene deletions were identified that either buffered cells against (synthetic‐rescue) or sensitized (synthetic‐sick‐lethal, SSL) to treatment with BIBR1532. Of the SSL interactions, we identified processes involved in pyrimidine salvage (e.g., *UCK2*), *de novo* pyrimidine synthesis (e.g. *DHODH*, *PDSS1*, *PDSS2*), and components of the INO80 chromatin remodeling complex (*INO80E*, *TFPT*, *NFRKB*, *UCHL5*; Figure 1e‐f, Table S1). These SSL relationships were recapitulated in cells deleted for the telomerase reverse transcriptase, *TERT* (*TERT* KO; Figure S1d‐g) using chemical inhibitors of the INO80 complex subunit UCHL5 (NSC‐687852 and WP‐1130), the nucleotide transporter SLC29A1 (NBMPR), and DHODH (atovaquone and brequinar; Figure S1h‐i). Given the known role of DHODH in pyrimidine biosynthesis (Evans & Guy, 2004), we tested if the reduced cell fitness observed in *TERT* KO cells treated with DHODH inhibitors could be rescued by nucleoside supplementation, and found that addition of exogenous nucleosides partially or completely rescued the sensitivity of *TERT* KO cells to atovaquone or brequinar, respectively (Figure S1j). These results are consistent with the known sensitivity of cells without telomerase to nucleotide pool homeostasis and replicative stress (Matmati et al., 2020), and with a previously reported role of INO80 in telomere replication, recombination and length homeostasis (Hu et al., 2013; Min et al., 2013; Morrison & Shen, 2009). Thus, the telomerase inhibitor BIBR1532 identified several genes whose function is known to be important to cell fitness when telomeres become eroded.

### 2.2 Genetic validation of a synthetic‐sick‐lethal interaction with *TAPR1* (*C16ORF72*) in cells lacking the telomerase reverse transcriptase

**FIGURE 2:**
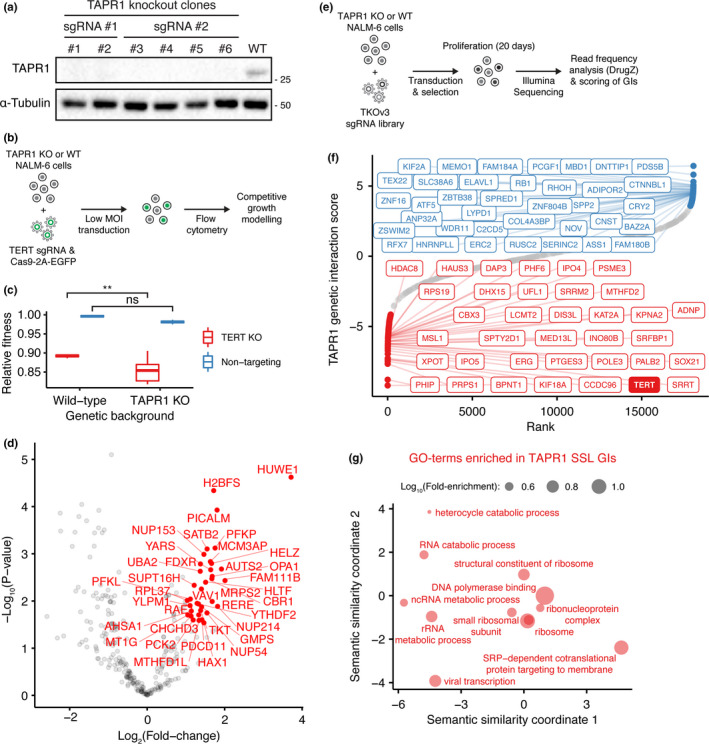
Analysis of TAPR1/*TAPR1* protein and genetic interactions. (a) NALM‐6 lysates from clonal *TAPR1*‐disrupted (TAPR1 KO) or wild‐type NALM‐6 cells were blotted against TAPR1 and α‐tubulin (1 representative blot of 2 independent replicates). (b) Schematic of competitive growth assays used to query the genetic interaction between *TAPR1* and *TERT*. (c) Relative fitness of *TERT*‐disrupted (TERT KO) or non‐targeting control in wild‐type or *TAPR1*‐deleted NALM‐6 cell background (*n* ≥ 3). (d) Volcano plot showing TAPR1 protein–protein interactions measured by BioID. Proteins with a peptide count fold‐change higher than 2 and a FDR lower than 0.1 are labeled in red (*n* ≥ 3). (e) Schematic of genome‐wide CRISPR screen in *TAPR1*‐deficient cells and genetic interaction scores. (f) Ranked *TAPR1* genetic interaction scores (see Appendix S1 for details). The top 1% SSL and buffering interactions (with the top 0.2% interactions labeled) are shown in red and in blue respectively. (g) Gene ontology (GO) term enrichment in the list of SSL genetic interactions with *TAPR1*. The position of GO terms represents their semantic similarity and a subset is labeled to aid visualization

One of the SSL interactions with BIBR1532 treatment was an unnamed gene, *C16ORF72* (Figure 1e, Table S1), hereafter referred to as *TAPR1* for Telomere Attrition and P53 Response 1. This gene encodes a predicted protein of 275 amino acids of unknown function that was also recently identified in a high‐throughput genetic screen as SSL with ATR inhibition (Hustedt et al., 2019). We disrupted *TAPR1* in NALM‐6 cells with two different sgRNAs (targeting exons 1 and 2) and isolated clones disrupted for *TAPR1* (*TAPR1* KO; Figure 2a; see Appendix S1 for details). Competitive growth experiments to assess the relative fitness of *TAPR1*‐deleted NALM‐6 cells versus NALM‐6 cells deleted for both *TAPR1* and *TERT* revealed a statistically significant decrease in cell fitness in cells lacking *TAPR1* and *TERT* (Figure 2b‐c, S2a,b). These data show that *TAPR1* exhibits a SSL interaction with cells in which telomerase function has been compromised.

### 2.3 Identification of physical and genetic interaction partners of TAPR1

To examine physical interactors of TAPR1, we performed protein proximity labeling (BioID) in NALM‐6 cells using TAPR1 as a bait (Figure 2d, see Appendix S1). The top TAPR1‐interactor was the E3 ligase HUWE1, which is known to mediate the ubiquitin‐dependent degradation of p53, MYC, and other substrates (Giles & Grill, 2020; Gong et al., 2020; Figure 2d, Table S2). Other TAPR1 interactors identified in the BioID analysis included proteins involved in proteostasis, the mRNA export machinery, and the nuclear pore, such as MCM3AP, NUP214, NUP153, NUP54, and RAE1 (Figures 2d, S2c, Table S2). To identify *TAPR1* genetic interactions, we performed a genome‐wide CRISPR screen in NALM‐6 cells disrupted for *TAPR1* and compared the cell fitness profiles to wild‐type NALM‐6 cells (Figures 2e, S2d). *TERT* was among the top SSL genetic interactions with *TAPR1* along with *ACD*, a gene that encodes the telomerase recruitment factor TPP1 (Figure 2f, S2d). GO‐term analysis of the list of *TAPR1* SSL genetic interactions revealed enrichment of genes involved ribosome biogenesis (Figure 2g, Table S3, see also Appendix S1). We compared the *TAPR1*‐/‐ genome‐wide screen data with *TAPR1* co‐dependencies in 769 different cell lines (Meyers et al., 2017; www.depmap.com). Genetic co‐dependencies with *TAPR1* were enriched in genes involved in ribosome biogenesis, replicative senescence, and p53 signal transduction in response to DNA damage (Figure S2e‐f). For example, genes with the highest co‐dependency included negative regulators of p53 activity such as *HUWE1*, *MDM2*, *MDM4*, *USP7*, and *PPM1D*. Genes with a negative co‐dependency included positive p53 effectors such as *TP53* itself, *TP53BP1*, *USP28*, *ATM*, *CHEK2*, and *CDKN1A* (p21). Several of these gene deletions also exhibited a buffering or SSL interaction with *TAPR1* loss, consistent with a role of these gene products as effectors or attenuators of p53, respectively (Figure S3). These data reveal that *TAPR1* exhibits a wide array of physical and genetic interactions that suggest a role in the p53 response. Importantly, our unbiased genome‐wide screen data indicate that a major genetic vulnerability of *TAPR1*‐deficient cells is the loss of telomere homeostasis.

### 2.4 The transcriptome of cells lacking *TAPR1* reveals signatures consistent with p53 signaling

**FIGURE 3:**
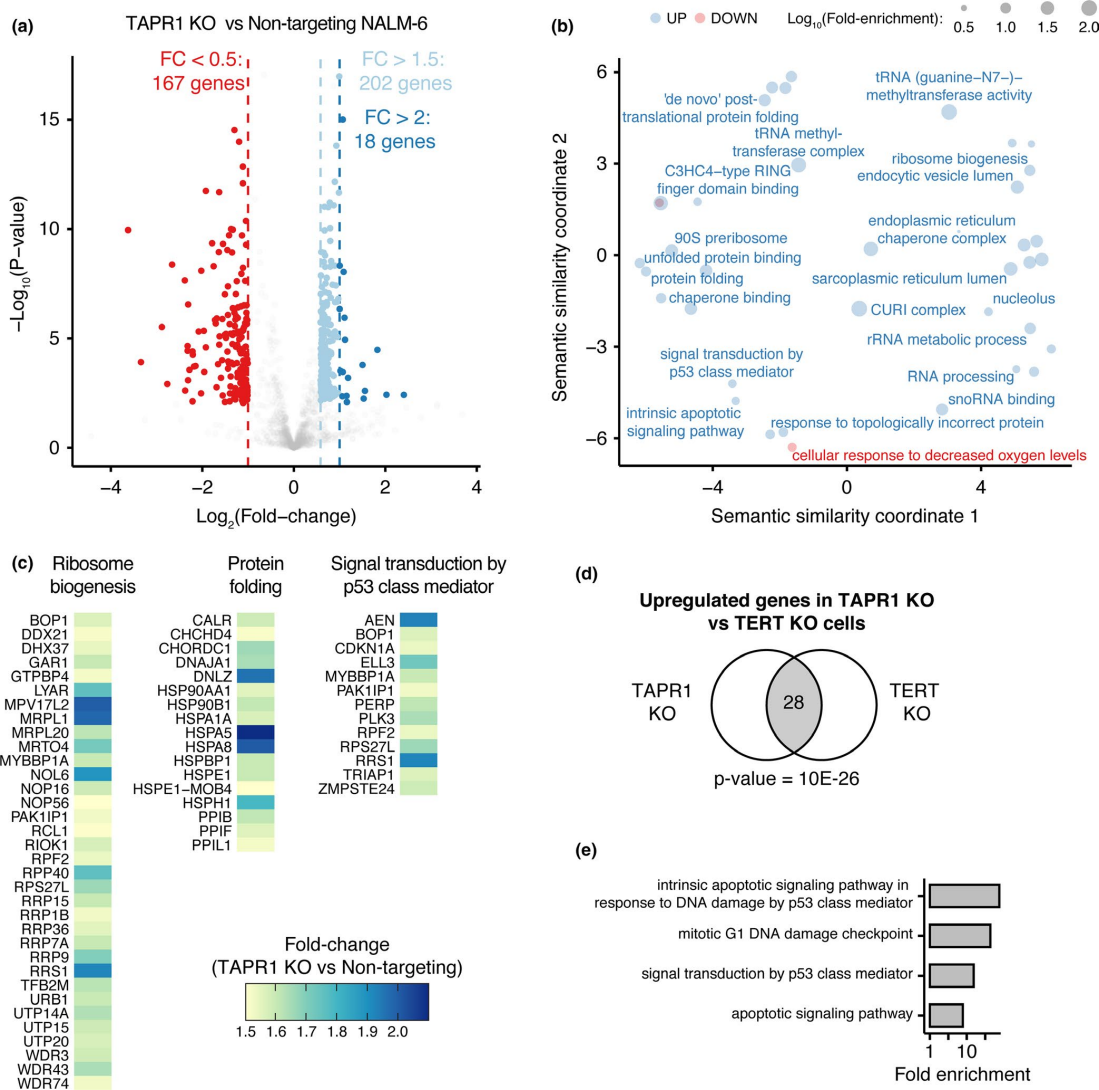
The transcriptome of cells lacking *TAPR1* exhibits upregulation of p53 signaling. (a) Volcano plot showing transcriptome changes in *TAPR1*‐disrupted (TAPR1 KO) NALM‐6 cells relative to non‐targeting controls, with differentially expressed genes (FDR < 0.05) shown for the fold‐change thresholds indicated (*n* ≥ 3). (b) Gene ontology (GO) term enrichment in the list of upregulated (fold change >1.5, shown in blue) or downregulated (fold change <0.5, shown in red) genes in TAPR1 KO NALM‐6 cells. The position of GO terms represents their semantic similarity and a subset is labeled to aid visualization. (c) Heatmap showing the fold change of upregulated genes within the indicated enriched GO terms. (d) Upregulated genes in cells lacking *TERT* (TERT KO) or *TAPR1* (TAPR1 KO) were used to calculate the statistical significance of the overlap (shown as number of genes in common in the gray‐shaded area) between the indicated lists of genes using the hypergeometric test. (e) GO‐term enrichment in the list of overlapping upregulated genes in NALM‐6 cells deleted for *TERT* or *TAPR1*

To further probe the potential relationship between TAPR1 and p53, we used RNA‐seq to assess the transcriptional response to deletion of either *TAPR1* or *TERT* compared with non‐targeted control cells (Figure 3a, Table S4). Of the gene networks upregulated in *TAPR1*‐deficient cells, we identified processes regulated by p53, as well as the response to proteotoxic stress via ribosome biogenesis (rRNA cleavage, SSU, and LSU assembly) and chaperone‐mediated protein folding (HSP90AA1, HSP90B1, HSPA1A, HSPA5, HSPA8, HSPE1, HSPH1, DNAJA1; Figure 3b‐c, Table S4). An analysis of the transcriptome of *TERT* KO NALM‐6 cells similarly detected an upregulation of p53‐regulated genes (Figure S4a‐b, Table S4), with a statistically significant overlap between the *TERT* KO and *TAPR*1 KO RNA‐seq datasets (Figure 3d‐e; see Appendix S1 for details). This upregulation of p53 transcriptional targets in *TERT* KO cells was accompanied by an increase in p53 levels (Figure S4c). These results suggest that *TAPR1* deficiency is associated with an upregulation of p53 and p53‐regulated genes, a subset of which are also observed in *TERT*‐deficient cells with eroded telomeres.

### 2.5 TAPR1 modulates p53‐mediated growth arrest

The upregulation of p53‐responsive genes in cells lacking *TERT* or *TAPR1* suggested that TAPR1 may be required to attenuate the p53 response as telomeres become eroded. To test this hypothesis, we examined if the stabilization of p53 had an adverse effect on NALM‐6 *TERT* KO cell fitness. We first confirmed that nutlin‐3a, which inhibits the interaction of p53 with MDM2 and thereby stabilizes p53 (Secchiero et al., 2011), led to the expected upregulation of p53‐dependent genes such as *BAX*, *CDKN1*, and *MDM2* (Figure S4d). We next tested the impact of nutlin‐3a treatment on the fitness of *TERT* KO cells, and observed that nutlin‐3a elicited a reduction in cell fitness compared with wild‐type cells (Figure S4e). This impact of nutlin‐3a on cell fitness in *TERT* KO cells was *TP53*‐dependent, since deletion of *TP53* rescued the reduction in cell fitness (Figure S4e). These data show that NALM‐6 cells exhibited a p53‐dependent reduction in cell fitness upon *TERT* loss.

**FIGURE 4:**
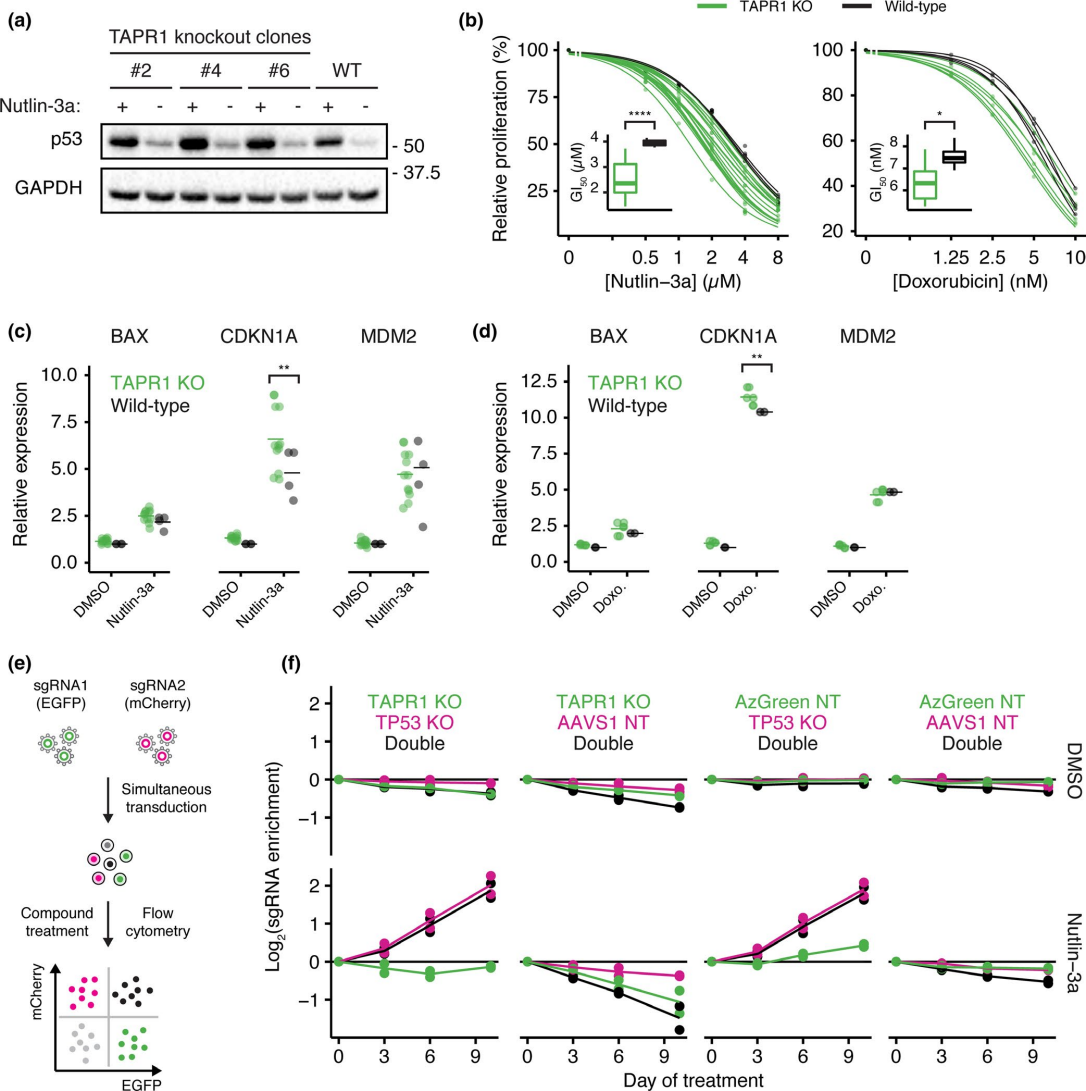
The impact of *TAPR1* loss on cell fitness is *TP53*‐dependent. (a) NALM‐6 lysates from clonal *TAPR1*‐disrupted (TAPR1 knockout) or wild‐type (WT) NALM‐6 cells treated with nutlin‐3a (2 µM, 4 h) were blotted against p53 and GAPDH (1 representative blot of 3 independent replicates). (b) Relative proliferation of *TAPR1*‐disrupted (TAPR1 KO) or wild‐type cells treated with the indicated concentrations of nutlin‐3a or doxorubicin for 72 h. Dose–response curves were fitted and the GI_50_ concentration is shown as inset plots (*n* ≥ 3). (c) Relative expression of the indicated transcripts in wild‐type or TAPR1 KO cells treated with 2 µM nutlin‐3a or 0.1% (v/v) DMSO for 4 h (*n* ≥ 4). (d) Relative expression of the indicated transcripts in wild‐type or TAPR1 KO cells treated with 0.5 µM doxorubicin (Doxo.) or 0.1% (v/v) DMSO for 4 h (*n* ≥ 2). (e) Competitive growth assay schematic for NALM‐6 cells transduced with non‐targeting sgRNAs and sgRNAs targeting *TAPR1* and *TP53*. (f) sgRNA enrichment in NALM‐6 cells treated with 2 µM nutlin‐3a or 0.1% (v/v) DMSO shown for the indicated *TAPR1*/*TP53* sgRNA combinations (*n* = 2)

In keeping with the transcriptional upregulation of p53‐dependent targets, we observed an increase in p53 protein levels in *TAPR1*‐deficient cells relative to wild‐type cells that was also apparent after nutlin‐3a treatment (Figure 4a). We also observed a decrease in relative fitness of *TAPR1*‐deleted cells upon treatment with nutlin‐3a or doxorubicin, a topoisomerase II poison that activates p53 in response to DNA damage (Figure 4b). These treatments led to a statistically significant upregulation of the p53 transcriptional target *CDKN1A* (Figure 4c‐d). These data suggested that the increased sensitivity of *TAPR1*‐deficient cells to nutlin‐3a and doxorubicin could be due to a p53‐mediated elevation of *CDKN1A*. We therefore tested whether disruption of *TP53* would rescue the sensitivity of *TAPR1*‐deficient cells to nutlin‐3a. Competitive growth assays in cells disrupted for *TAPR1* or *TP53* alone, or both genes deleted together (Figure 4e, see Appendix S1), revealed that the deletion of *TP53* was epistatic to deletion of *TAPR1* with respect to nutlin3a sensitivity (Figure 4f; compare first and third lower graphs). These data are consistent with a role of TAPR1 in buffering against the deleterious effect on cell fitness when p53 is activated by eroded telomeres, DNA damage or MDM2 inhibition.

## 3 DISCUSSION

We conducted a genome‐wide screen using CRISPR‐Cas9 in the p53‐positive cancer cell line, NALM‐6, for gene deletions that sensitized cells to telomere erosion. We uncovered a previously unannotated gene, *C16ORF72*, which we named *TAPR1* (Telomere Attrition and P53 Response 1) as it exhibits a strong synthetic genetic interaction with telomerase inhibition or deletion of *TERT*, and appears to taper the response to p53 upon loss of telomere integrity.

Prior to this study, *C16ORF72*/*TAPR1* was identified as a hit in human genome‐wide CRISPR‐Cas9 chemogenetic screens for sensitizers to ATR kinase inhibition or hydroxyurea (Hustedt et al., 2019; Benslimane et al., 2020). *C16ORF72*/*TAPR1* is also a target of miR‐134, a microRNA associated with tumorigenesis and chemo‐resistance (Shuang et al., 2015), and is associated with rare *de novo* CNVs in autism spectrum disorder (ASD) and schizophrenia (Levinson et al., 2011; Sanders et al., 2011). In our study, TAPR1 was identified as an interactor with HUWE1, and C16ORF72/TAPR1 also interacts with HUWE1 in previous high‐throughput mass spectrometry surveys (Hein et al., 2015; Thompson et al., 2014). This HUWE1‐TAPR1 interaction may be biologically relevant, especially as p53 is ubiquitinated by HUWE1 (Giles & Grill, 2020; Gong et al., 2020). This interaction could be mediated by a domain of unknown function within TAPR1 (DUF4588), as the *S. cerevisiae* homologue of HUWE1 (Tom1) interacts with a protein of unknown function called YJR056C that contains the same DUF4588 domain (Sung et al., 2016). Tom1 and HUWE1 are involved in the degradation of ribosomal (ERISQ pathway) and non‐ribosomal proteins (Sung et al., 2016; Xu et al., 2016), and in the regulation of genes involved in ribosomal biogenesis (Gomez‐Herreros et al., 2017). HUWE1 is also implicated in the p53 response to proteotoxic stress caused by the imbalance between ribosomal protein (RP) and ribosomal RNA (rRNA) production (Hipp et al., 2019). We found several genes involved in proteostasis that were upregulated in *TAPR1*‐deficient cells, including components of the CURI complex that coordinate the HSF1‐dependent response to the imbalance between RPs and rRNA, subunits of the ribosomal SSU and LSU processosome, and other transcriptional targets of HSF1 (Figure 3a, Table S4). Future studies will determine if TAPR1 functions with HUWE1 in these ubiquitin‐mediated processes, and their relationship to the response to telomere erosion and DNA damage.

Our choice of a p53‐positive cell line for the CRISPR‐Cas9 screen enabled the identification of *TAPR1* as a gene whose function enables NALM‐6 cells to better cope with telomere loss via the attenuation of p53 activation. This result is supported by our unbiased genome‐wide screen data that identified deletion of *TERT* or *ACD* (encoding TPP1) as SSL with *TAPR1* loss. The role of TAPR1 may not be limited to a telomere‐induced DNA damage response (DDR), as *TAPR1*‐deficient cells were sensitive to nutlin‐3a, doxorubicin, and ATR inhibition (Hustedt et al., 2019; this study), and HUWE1 is also involved in the DDR (Hall et al., 2007; Mandemaker et al., 2017; Myant et al., 2017 Guo, 2020 #299). Intriguingly, the profile of genetic interactions also hint that TAPR1 might link proteotoxic stress and genome/telomere integrity, especially as aneuploidy is known to induce proteotoxic stress due to gene copy number imbalance (Brennan et al., 2019; Ohashi et al., 2015; Oromendia et al., 2012).

**FIGURE 5:**
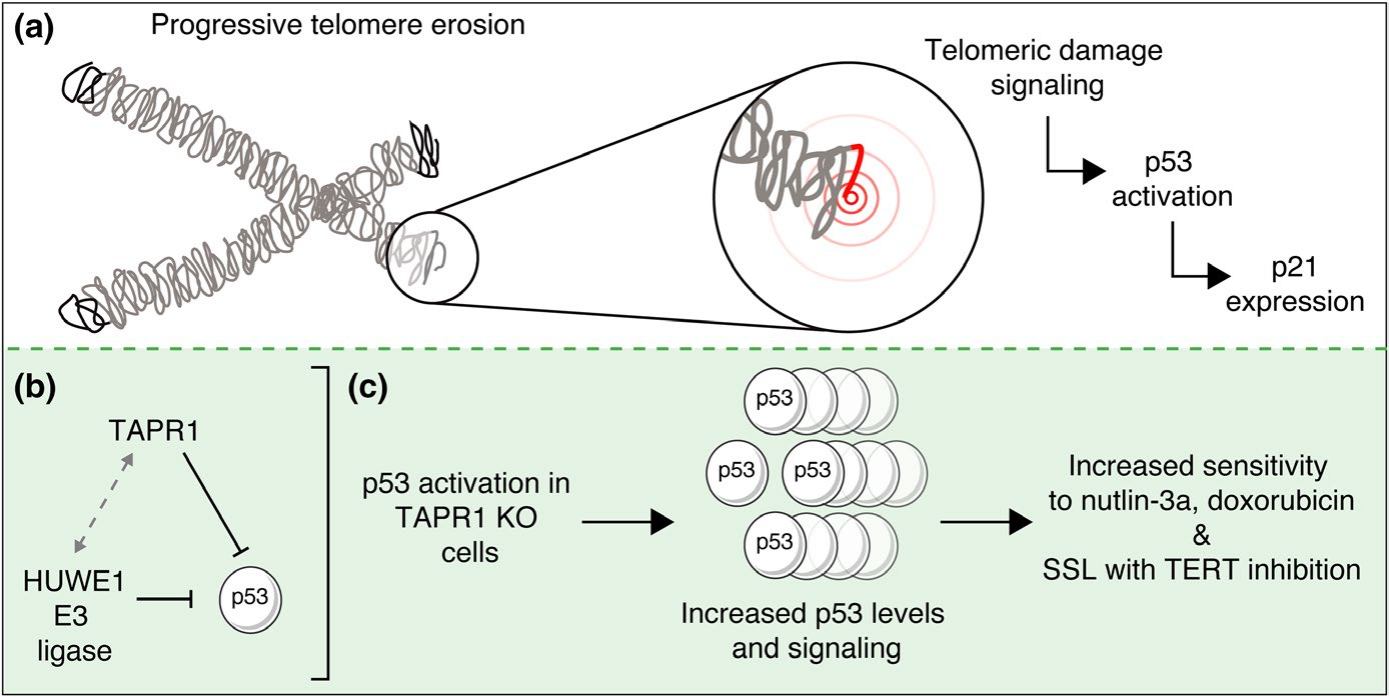
Model of TAPR1 modulation of p53 signaling in response to telomere erosion. (a) In the absence of sufficient telomere‐replenishing activity (e.g. telomerase inhibition), telomeres progressively erode and eventually induce a DNA damage response, resulting in p53 activation and induction of transcriptional targets such as *CDKN1a*/p21. (b) TAPR1 attenuates p53 activation in the pre‐B cell line NALM‐6. Proximity labeling identified HUWE1, an E3 ubiquitin ligase that targets p53 for degradation, as an interaction partner with TAPR1. Whether TAPR1 attenuates p53 in a HUWE1‐dependent manner has not yet been determined. (c) Deletion of TAPR1 leads to excessive p53 induction and increased sensitivity of cells treated with the MDM2 inhibitor nutlin‐3a or the DNA damaging agent doxorubicin, and a synthetic/sick/lethal (SSL) phenotype in cells either deleted for telomerase (TERT) or treated with the telomerase inhibitor BIBR1532. TAPR1 may also limit p53 activation in other circumstances including DNA damage, senescence and cancer

Cells must optimize the p53 response to satisfy two countervailing forces: too little p53 activity allows the propagation of damaged genomes that sets cells on the road to tumorigenesis, whereas too much p53 activity can drive cells into premature senescence (Wu & Prives, 2018). Because p53 lies at the nexus of cancer and aging, its appropriate regulation necessitates many context‐specific checks and balances that shape its overall activity (Sharpless & DePinho, 2002). Notably, a gradient of p53 activity appears to govern stem cell fitness in part through cell non‐autonomous competition effects that are manifest at lower levels of p53 activation (Bondar & Medzhitov, 2010). Our identification of TAPR1 as an attenuator of p53 in the context of low level genomic damage caused by eroded telomeres adds a new element to the p53 regulatory network (Figure 5). Given the role of p53 activation as a key mediator of senescence (Wu & Prives, 2018), it will be important to determine how *TAPR1* expression is regulated in primary cells as telomeres erode, and if *TAPR1* serves to protract the entry into senescence in normal cells and/or tissues by counterbalancing p53 activation. In mice with eroded telomeres, p53 deficiency initially rescues the adverse effects of telomere dysfunction on proliferation but then promotes subsequent tumor initiation (Chin et al., 1999). *TAPR1* may thus play a role in delaying cellular senescence on the one hand and suppressing tumorigenesis or apoptosis in response to genotoxic stress on the other hand. Regardless of its specific physiological roles, TAPR1 represents a previously unappreciated genetic modulator of the delicate equilibrium that governs p53 activity. Future work will illuminate the precise genetic and cellular contexts in which TAPR1 is important for p53 function and other biological responses to stress, and how these functions impinge on cancer and aging.

## 4 EXPERIMENTAL PROCEDURES

### 4.1 Cell culture

NALM‐6 cells are an immortalized cell line that was established from the peripheral blood of a man, 19 years of age, with acute lymphoblastic leukemia (see Key Resources in Appendix S1) and was provided by the laboratory of Steve Elledge. Cells were propagated in 10% FBS (v/v) RPMI 1640 medium and 37°C, and sub‐cultured every 2–3 days. HEK293 T cells (for lentiviral packaging) were propagated in 10% FBS (v/v) DMEM medium at 5% (v/v) CO_2_ and 37°C, and sub‐cultured every 2–3 days. Parental and modified (e.g. knockout) cell lines used for this study were tested for mycoplasma contamination by standard multiplex PCR. The genotype of knockout lines (populations or clonal isolates) were confirmed by indel sequencing (see Appendix S1). Further details on generation of knockout lines, proliferation assays and competitive growth modeling are provided in Appendix S1.

### 4.2 Genome‐wide CRISPR screens

The CRISPR knockout screen to identify chemical‐genetic interactions with BIBR1532 was performed as previously described with the following changes (Benslimane et al., 2020). A NALM‐6 clone with inducible Cas9 expression previously transduced with the EKO library was treated with Doxycycline (2 µg/mL) for 8 days to induce Cas9 expression and knockout generation followed by treatment (28 million cells per treatment, corresponding to 100 cells/sgRNA) with 20 µM BIBR1532 or 0.1% (v/v) DMSO for 20 days. For the genome‐wide CRISPR screen in *TAPR1*‐deficient cells, wild‐type NALM‐6 cells or two independent clones of TAPR1‐disrupted NALM‐6 cells were transduced with the TKOv3 sgRNA library as previously described (Benslimane et al., 2020; Hart et al., 2017). Briefly, 120 million cells were transduced at a MOI of 0.5, corresponding to a coverage of 800 cells/sgRNA. Two days after infection, cells were selected with puromycin (0.5 µg/mL) for 4 days. After selection, 36 million cells from each of the cell populations harboring the TKOv3 libraries proliferated for an additional 15 days (with sub‐culturing every 3 days). After the sgRNA library outgrowth period (for both genome‐wide screens performed), cells were collected, genomic DNA extracted and sgRNA sequences were PCR‐amplified followed by Illumina sequencing (Illumina NextSeq500) at the Genomic Platform at Institute for Research in Immunology and Cancer (IRIC). Reads were aligned using Bowtie2.2.5 in the forward direction only (‐‐norc option) with otherwise default parameters and total read counts per sgRNA tabulated to obtain sgRNA frequencies. For the DMSO controls, sgRNA sequencing reads from multiple DMSO‐treated samples were pooled as background, to decrease noise. Chemical‐genetic interactions were scored relative to DMSO using the previously published RANKS algorithm (Bertomeu et al., 2017), which estimates the *p*‐values for the fold change between individual sgRNAs to control sgRNAs (non‐targeting sgRNAs in the EKO library) or the DrugZ algorithm (see Appendix S1, Key Resources). The *TAPR1* genetic interaction data was analyzed using the DrugZ algorithm by comparing the sgRNA read frequency in each *TAPR1*‐deleted NALM‐6 clone against the summed reads from two replicates in a wild‐type NALM‐6 background. The NormZ scores for each clone were subsequently summed to generate a TAPR1 genetic interaction (GI) score to highlight genes that show a SSL or buffering phenotype in both *TAPR1*‐deleted clones.

### 4.3 Transcriptome analysis by RNA‐Seq

RNA from 1 million clonal NALM‐6 cells of the indicated genotypes was extracted using the QIAGEN Mini RNeasy kit according to the manufacturer's protocol. Sample purity was assessed by nanodrop using 260/280 nm and 260/230 nm ratios. Total RNA was quantified by QuBit (ABI) and 1 µg of total RNA was used for library preparation. RNA quality control was assessed with the Bioanalyzer RNA 6000 Nano assay on the 2100 Bioanalyzer system (Agilent technologies) and all samples had a RIN of 10. Library preparation was performed with the KAPA mRNAseq Hyperprep kit (KAPA, #KK8581). Libraries were quantified by QuBit and BioAnalyzer and diluted to 10 nM before normalization by qPCR using the KAPA library quantification kit (KAPA; #KK4973). Libraries were then pooled to equimolar concentration. Sequencing was performed with the Illumina Nextseq500 on half a flowcell of Nextseq 75 cycles High Output v2 using 2.8 pM of the pooled libraries. Around 20 million single‐end PF reads were generated per sample. Analysis of RNA‐seq reads is described further in Appendix S1.

### 4.4 Gene list enrichment and network analysis

Statistical significance of the overlap between gene lists was calculated using the hypergeometric test in R. Gene ontology term enrichment was calculated with the “gprofiler2” package in R where the gene lists were considered an unordered query and with subsequent filtering for GO terms (GO:BP, GO:CC, GO:MF as data sources) that contain <1000 terms with an adjusted *p*‐value below .05 (see Appendix S1, Key Resources). Further filtering of the enriched GO terms was performed to remove redundant GO terms that share a high semantic similarity using the REVIGO tool (http://revigo.irb.hr/) to aid with visualization. The network of *TAPR1* co‐dependencies was built using the Avana CRISPR dataset (Public release 20Q3). This dataset is available as a matrix of normalized dependency scores that represents the effect of a CRISPR knockout in a given cell line. By calculating a correlation matrix using the Pearson method, co‐dependencies with a given gene knockout (most highly correlated or anti‐correlated genes) can be retrieved. The top 100 co‐dependencies with *TAPR1* (absolute Pearson correlation coefficient) as well as their respective top 10 co‐dependencies were combined (as nodes, with edges representing the co‐dependency). The network shown in Figure S3 was built with isolated nodes (i.e. genes with only 1 co‐dependency) removed for visual simplicity. The network was built and visualized as an undirected force‐directed layout (with minimal manual adjustments were made to improve legibility) using the “network”, “ggnetwork,” and “ggplot2” packages in R. The nodes in the network are color‐coded based on the score in the TAPR1 genetic interaction screen.

### 4.5 Quantification and statistical analysis

Unless otherwise indicated, statistical analyses were performed on PRISM 8 (www.graphpad.com). Statistical significance was carried out with a Student *t* test (2 groups), or with ANOVA (more than 2 groups) using the Sidak or Tukey correction for multiple comparisons.

## CONFLICT OF INTEREST

The authors declare no competing interests.

## AUTHOR CONTRIBUTIONS

Y.B., M.T., and L.H. conceptualized the study. Y.B., M.S.‐O., J.C.‐H., T.B., D.H. C.H., and E.B. carried out the methodology and investigation. Y.B. and L.H. wrote the manuscript with editorial input from all authors. P.T., M.T., and L.H. acquired funding and supervised the study.

## Supporting information

Appendix S1

Table S1

Table S2

Table S3

Table S4

## Data Availability

All unique/stable reagents generated in this study will be made available upon reasonable request. Depending on the reagent, a completed materials transfer agreement may also be required. Further information and requests for resources and reagents should be directed to and will be fulfilled by the Lead Contact, Lea Harrington ( lea.harrington@umontreal.ca ). CRISPR screen HTS sequencing and RNA‐Seq data are uploaded and available at NCBI GEO https://www.ncbi.nlm.nih.gov/geo/query/acc.cgi?acc=GSE160869. The mass spectrometry proteomics data (BioID) have been deposited to the ProteomeXchange Consortium via the PRIDE partner repository with the dataset identifier PXD022128 and 10.6019/PXD022128 (see also Appendix S1, Key Resources).
