# Supplementary material for: A novel p53 regulator, C16ORF72/TAPR1, buffers against telomerase inhibition
Source: Aging Cell. 2021 Mar 4;20(4):e13331. doi: 10.1111/acel.13331 (PMC8045932; doi:10.1111/acel.13331)
Supplement: Supplementary file 1 — Appendix S1 [file ACEL-20-e13331-s004.pdf]

<u>Benslimane et al., Supporting information</u>

Supplementary Table 1, **related to figure 1**: BIBR1532 CRISPR screen gene scores analyzed by the RANKS or DrugZ algorithms.

Supplementary Table 2, related to figure 2: TAPR1-interacting proteins identified by proximity-labeling in NALM-6 cells (BioID).

Supplementary Table 3, related to figure 2: *TAPR1* genetic interactions identified by genome-wide CRISPR screening in *TAPR1* KO NALM-6 cells.

Supplementary Table 4, related to figure 3: Differentially expressed genes in *TAPR1*- or *TERT*-disrupted NALM-6 cells measured by RNA-Seq and analyzed by the DESeq2 algorithm.

Benslimane et al., Figure S1

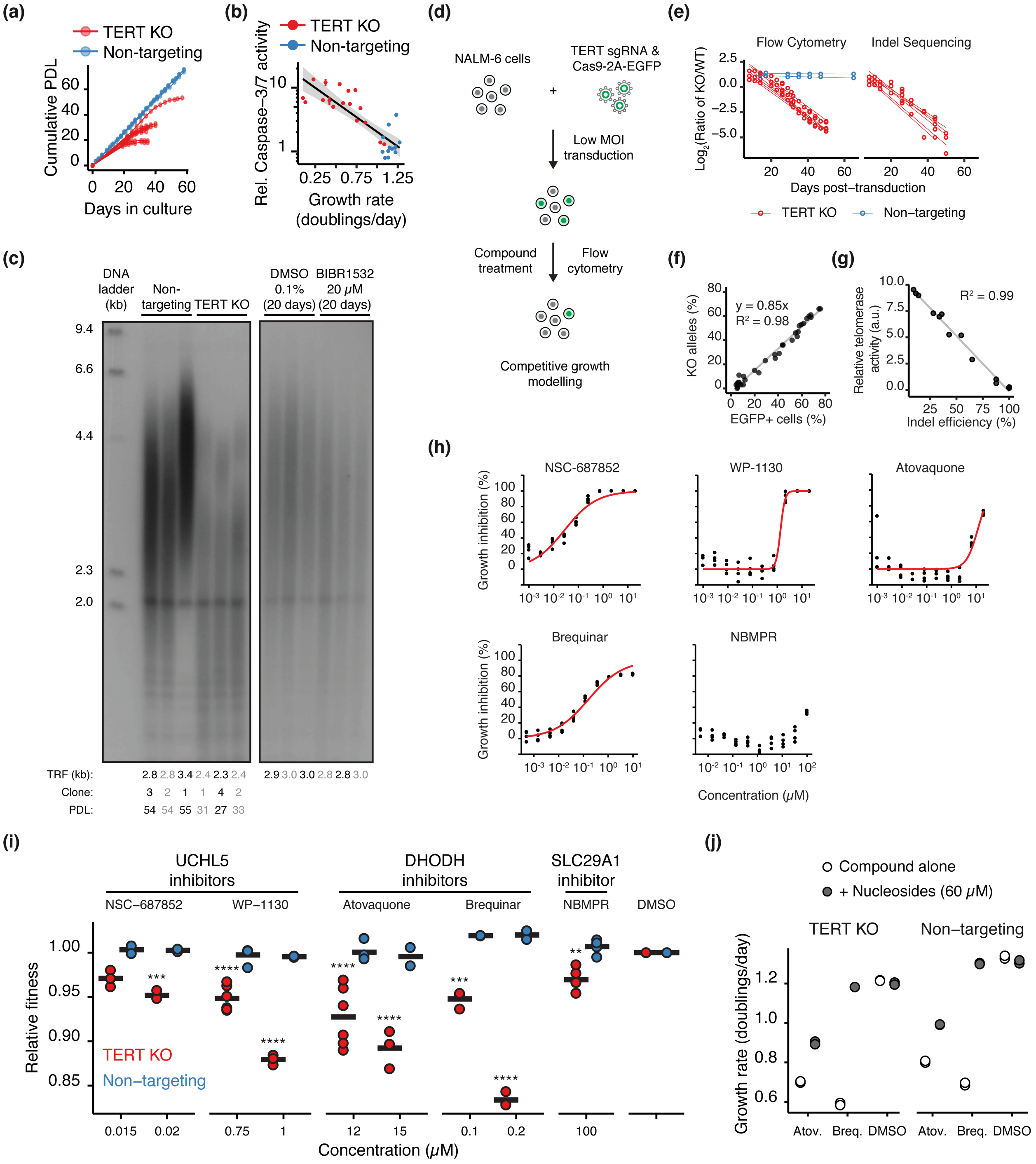

Figure S1: Genetic and chemical sensitivities of NALM-6 cells to *TERT* disruption or telomerase inhibition, related to figure 1. (a) Cumulative population doublings relative to days in culture in clonal *TERT*-disrupted (TERT KO; n=8) or non-targeting control NALM-6 cells (n=3). (b) Relative Caspase-3/7 activity in *TERT*-disrupted cells (TERT KO; n=4) or non-targeting (n=3) NALM-6 cell lysates relative to the growth rate at the time of lysate collection (n=3 technical replicates per sample). (c) TRF measurement of telomere length. Left, clonal *TERT*-disrupted (TERT KO) or non-targeting NALM-6 cells (PDL indicated below respective lanes); right, NALM-6 cells treated with BIBR1532 or DMSO for 20 days (n=3). Mean TRF length is quantified and indicated below each lane. (d) Competitive growth assay schematic in NALM-6 cells used to measure the relative fitness of cells lacking *TERT* (KO) under different conditions. (e) Log-transformed ratio of KO to WT cells as measured by flow cytometry or indel sequencing for mixed populations of TERT KO (n=6) or non-targeting (n=2) cells. (f) A mixed population of cells targeted for *TERT* (measured by Sanger sequencing) relative to the transduction efficiency (GFP+ cells, measured by flow cytometry). (g) Relative telomerase activity of NALM-6 cell lysates measured by qTRAP relative to the indel efficiency in the mixed cell populations. h. Growth inhibition of NALM-6 cells treated with the indicated compounds for 72 h (n=4). (i) Relative fitness of *TERT*-disrupted (TERT KO; n ≥ 3) or non-targeting (n ≥ 2) mixed populations treated with the indicated compounds. (j) Growth rate of *TERT*-disrupted (TERT KO) or non-targeting non-clonal populations treated with atovaquone (12 µM), brequinar (0.1 µM) or DMSO (0.1% v/v) with addition of nucleosides in the media (n=2).

Benslimane et al., Figure S2

(a)

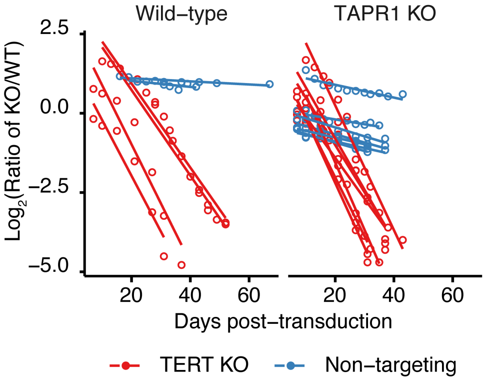

(d)

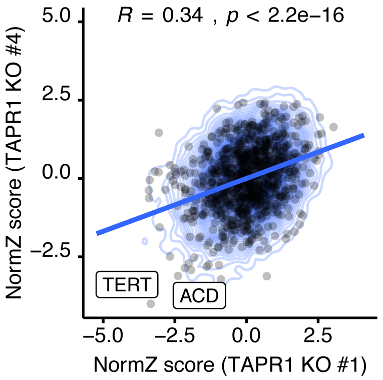

(b)

Gene targeted and clone number	Frameshift genotype
TERT KO, clone #1	(+5, +7)
TERT KO, clone #2	(+1, +2)
TERT KO, clone #3	(+4, +2)
TERT KO, clone #4	(+7, -4)
TAPR1 KO, clone #1	(+1, +5)
TAPR1 KO, clone #2	(+2, -11)
TAPR1 KO, clone #3	(+5, +5)
TAPR1 KO, clone #4	(+2, +2)
TAPR1 KO, clone #5	(-1, +4)
TAPR1 KO, clone #6	(+2, +4)

(e)

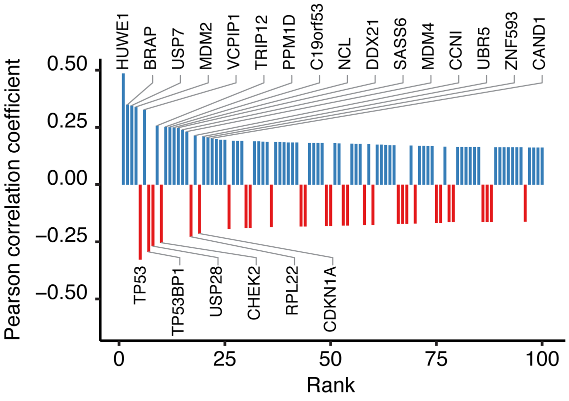

(c)

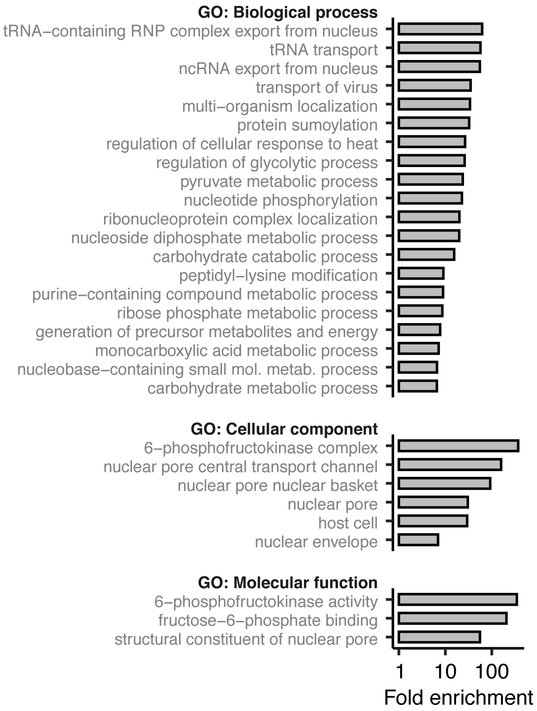

(f)

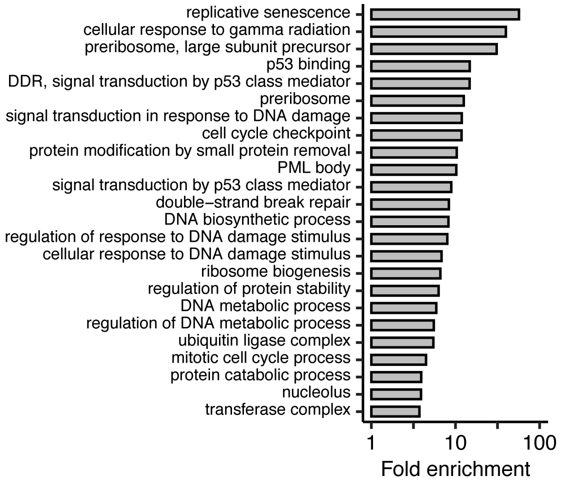

Figure S2: Cell proliferation of NALM-6 cells disrupted for *TERT* or *TAPR1*, and Gene Ontology (GO) enrichment of TAPR1 genetic or protein interactions, related to figure 2. (a) Flow cytometry measurement of EGFP-positive *TERT*-disrupted (*TERT* KO) or non-targeting cells relative to the days post-transduction of *TAPR1*-disrupted (*TAPR1* KO) or wild-type NALM-6 cells (n ≥ 2). (b) Indel genotyping of frameshifts in the clonal populations generated for *TERT* and *TAPR1* KO cells. (c) GO-term enrichment in the list of TAPR1 protein interactors identified by BioID. (d) Pearson correlation of the *TAPR1* genetic interaction scores in both clonal backgrounds used in the double-KO genome-wide screen. A sample of points used to calculate the correlation is shown to aid in visualization, and the points that correspond to *TERT* and *ACD* are highlighted. (e) Top 100 correlates with *TAPR1* for genetic dependency in the AVANA dataset (DepMap). Gene labels indicate correlates with an absolute value of the Pearson correlation higher than 0.2. (f) GO-term enrichment in the top 100 correlates with *TAPR1* for genetic dependency in the AVANA dataset (DepMap).

Benslimane et al., Figure S3

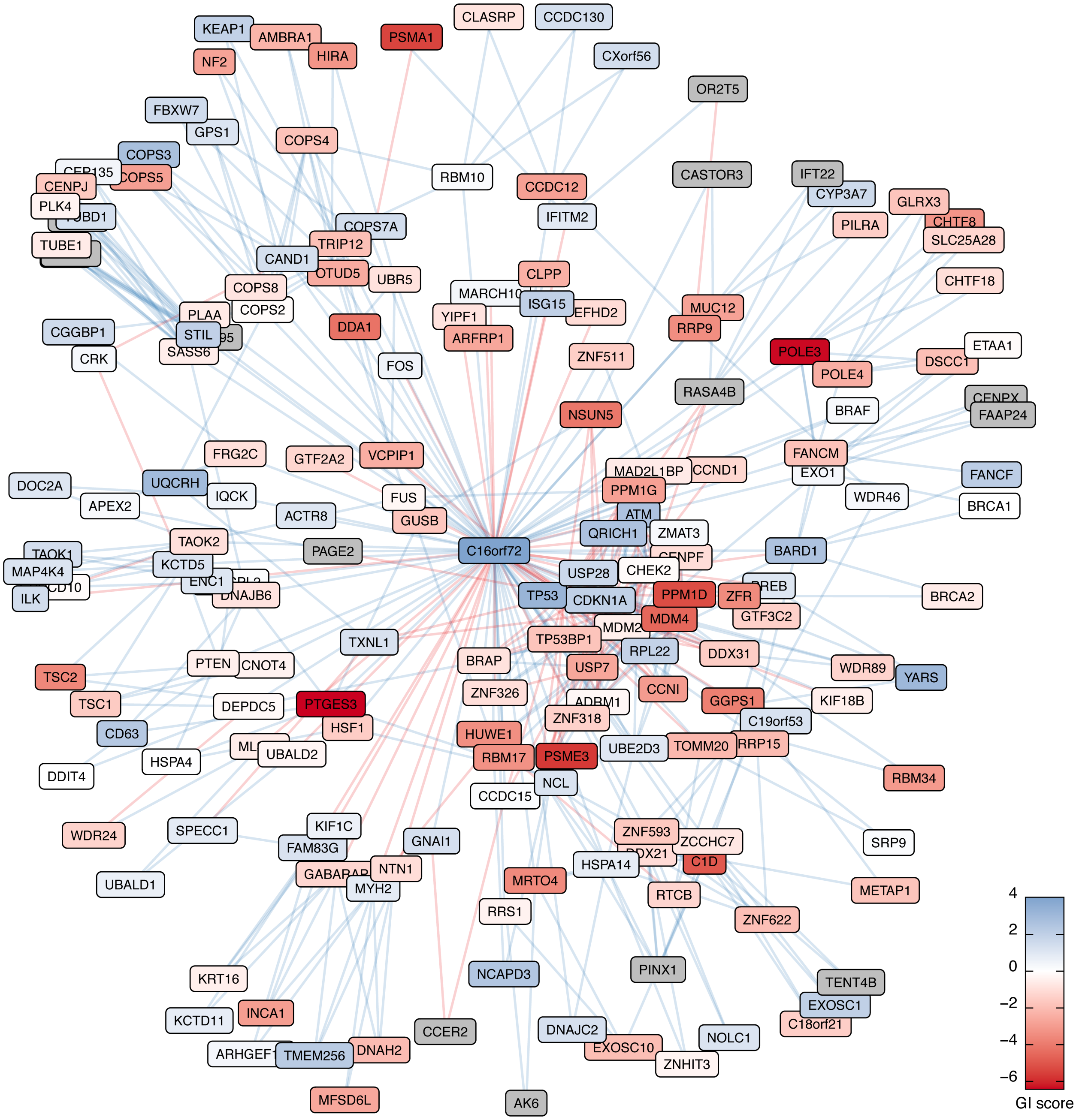

Figure S3: Schematic of *TAPR1* co-dependencies as identified via DepMap, related to figure 2. The top 100 co-dependent genes with *TAPR1* are shown, including the top 10 co-dependencies of each of these 100 genes. Edge color represents the DepMap co-dependency Pearson correlation coefficient direction (blue: positive correlation, red: negative correlation). Node color represents the genetic interaction (GI) score as measured in the *TAPR1* knockout CRISPR screen, and grey nodes indicate genes that were not scored in the CRISPR screen.

Benslimane et al., Figure S4

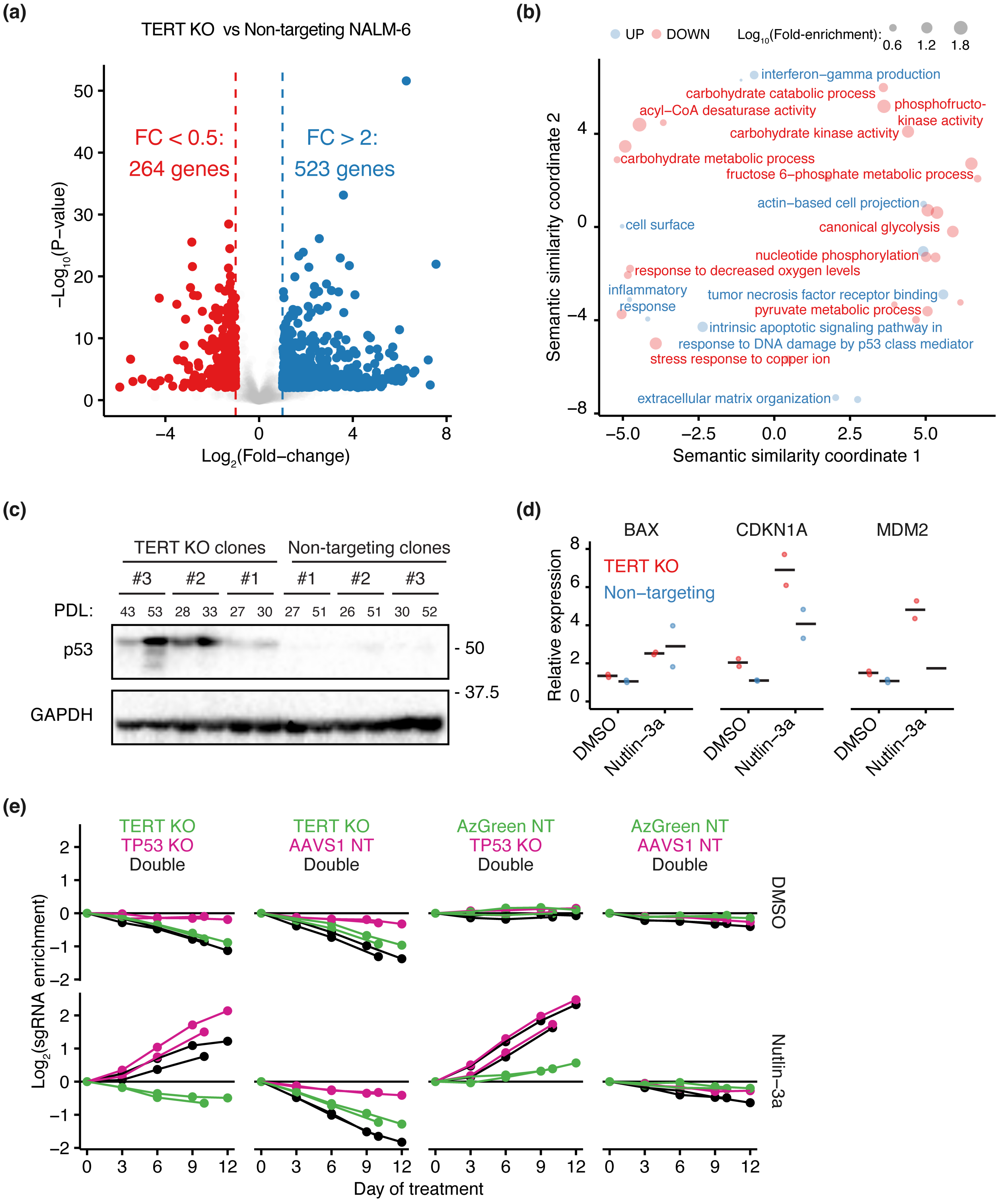

Figure S4: RNA-seq analysis of *TAPR1*- and *TERT*-deficient NALM-6 cells, related to figure 3. (a) Volcano plot showing transcriptome changes in *TERT*-disrupted (TERT KO) NALM-6 cells approaching crisis relative to non-targeting controls; differentially expressed genes (FDR < 0.05) are shown for the different fold-change cutoffs (n=3). (b) Gene ontology (GO) term enrichment in the list of upregulated (fold-change > 2, shown in blue) or downregulated (fold-change < 0.5, shown in red) genes in TERT KO NALM-6 cells. The position of GO-terms represents their semantic similarity and a subset is labeled to aid with visualization. (c) Lysates from NALM-6 cells disrupted for *TERT* (TERT KO) or non-targeting controls collected at different timepoints after clonal selection (population doubling [PDL] at the time of collection is indicated above respective lanes) were blotted against p53 and GAPDH (1 representative blot of 2 independent replicates). (d) Relative expression of the indicated transcripts in *TERT* KO cells or non-targeting controls (after EGFP-positive cell sorting from population knockouts) treated with 2 µM nutlin-3a or 0.1% (v/v) DMSO for 6 days (n=2). (e) sgRNA enrichment in NALM-6 cells treated with 2 µM nutlin-3a or 0.1% (v/v) DMSO for the indicated TERT/TP53 sgRNA combinations (n=2).

SUPPLEMENTARY METHODS

Proliferation assays

Compounds (please refer to Key resources for details) were re-suspended in DMSO and serially diluted 3-fold (or 4-fold for BIBR1532) and added to their respective wells in 384-well plates along with 9000 NALM-6 cells in a final volume of 50 µL (0.1% [v/v] DMSO). Plates were incubated for 72h at 37 °C before addition of the CellTiter-Glo reagent and luminescence measurement. Relative proliferation was calculated as a ratio of average luminescence of the treatment to the average luminescence of the DMSO controls and subtracted from 100% to obtain growth inhibition. Low-throughput proliferation assays were performed by seeding NALM-6 cells at 1 x 10^5 cells/mL in 24-well plates and compounds were added at a 1:1000 dilution to yield a final DMSO concentration of 0.1% (v/v). After 72 h of incubation at 37 °C, cell concentration was measured on a Beckman-Coulter Z2 Counter after debris exclusion by particle size gating. Relative proliferation was calculated as a ratio of the population doubling of the treatment to the population doubling of the respective DMSO control. Dose-response curves were fitted as 4-parameter nonlinear regression using R (with lower bound constrained at 0 and upper bound constrained at 100) and the half-maximal growth inhibition (GI_{50}) extracted from the fitted curves.

Targeted gene disruption using CRISPR-Cas9

Gene disruptions in NALM-6 cells were carried out using CRISPR-Cas9 editing as described previously (Benslimane et al., 2020). Briefly, sgRNAs targeting a given gene were designed using "sgRNA designer" tool (https://portals.broadinstitute.org/gpp/public/analysis-tools/sgrna-

design) (Doench et al., 2016) before cloning into LentiCRISPRv2 (a gift from Feng Zhang; Addgene #52961) (Sanjana et al., 2014), LentiCRISPRv2GFP (a gift from David Feldser; Addgene #82416) (Walter et al., 2017) or LentiCRISPRv2-mCherry (a gift from Agata Smogorzewska; Addgene #99154) as described in Sanjana et al. (Sanjana et al., 2014). The resulting plasmids were purified and sequence-verified to confirm the correct sgRNA sequence was inserted downstream of the U6 promoter before lentiviral packaging in HEK293T using the psPAX2 (a gift from Didier Trono; Addgene #12260) and pCMB-VSV-G plasmids (a gift from Bob Weinberg; Addgene #8454) (Stewart et al., 2003). Lentiviral transduction was performed by incubating viral particles with protamine sulfate (10 µg/mL) and 10^6 NALM-6 cells in a final volume of 2 mL for 48 h at 37 °C. The percentage of EGFP-positive and mCherry-positive cells were measured on a BD FACSCanto II or BD LSRFortessa after FSC/SSC gating to calculate transduction efficiency. Clonal isolates of *TERT*-disrupted (*TERT* KO) cells or non-targeting controls were isolated by single cell sorting on a BD FACSAria II from NALM-6 cells transduced with LentiCRISPRv2-Puro expressing TERT or AAVS1 targeting sgRNAs after puromycin selection. Clonal *TAPR1*-disrupted cell isolates (*TAPR1* KO) were isolated by single cell sorting after nucleofection (Lonza 4D Nucleofector, Lonza #AAF-1002B) and overnight incubation of NALM-6 cells with the respective sgRNA-expressing LentiCRISPRv2GFP plasmids with the SF Cell Line 4D-Nucleofector X Kit L. Indel efficiency and clonal genotyping was performed on genomic DNA using target locus PCR amplification and Sanger sequencing. The ICE online tool (https://ice.synthego.com/) was used for indel decomposition of the sequencing traces using the TIDE method (Brinkman et al., 2014). Indel efficiency for each knockout population was calculated as the indel quantification by TIDE

normalized by the EGFP+ percentage as a proxy for transduction efficiency. The genotype of each clonal population was inferred directly from the sequencing chromatogram indel decomposition.

Quantitative telomerase repeat amplification protocol (qTRAP)

Telomerase activity measurement was performed as previously described with minor modifications (Herbert et al., 2006). Briefly, 1 million NALM-6 cells were lysed in 100 µL of CHAPS buffer (10 mM Tris-HCl pH 7.5, 1 mM $MgCl_2$, 1 mM EGTA, 0.1 mM Benzamidine, 5 mM β-mercaptoethanol, 0.5% (w/v) CHAPS, 10% (v/v) glycerol) and protein quantification was performed using the Bradford assay. On the day of the assay, all cell lysates were diluted 1:10 in CHAPS buffer and NALM-6 cell standard curves were subsequently prepared by 2-fold serial dilutions. qPCR reactions were prepared in triplicate using the FastStart SYBR Green 2X mastermix, 1 mM EGTA, 0.8 µM ACX primer, 0.8 µM TS primer and 2 µL of cell lysate (1000 cells/µL) in a final volume of 25 µL and incubated in the StepOnePlus thermocycler (Applied Biosystems) with the following program (30 min at 30 °C, 10 min at 95 °C, 40 cycles of 15 sec at 90 °C and 60 sec at 60 °C). Analysis of Relative Telomerase Activity (RTA) was performed by averaging the C_T in technical replicates and using the average C_T as x-values in the NALM-6 standard curve to retrieve the telomerase activity as cell number equivalent followed by normalization to protein concentration. For the measurement of telomerase activity inhibition in NALM-6 WT cells, BIBR1532 dilutions were prepared as 2.5% (v/v) DMSO working solutions and added at a final DMSO concentration of 0.2% (v/v) to reach the indicated BIBR1532 concentrations in the qPCR reactions. Analysis of Relative Telomerase Activity (RTA) was performed by averaging the C_T in technical replicates and using the average C_T as x-values in the

NALM-6 standard curve to retrieve the telomerase activity as cell number equivalent for each BIBR1532 concentration divided by the DMSO control. The RTA data was fitted with a 4-parameter nonlinear regression with GraphPad Prism 8 to obtain the dose-response curve and half-maximal inhibitory concentration (IC_{50}).

Caspase-3/7 activity measurement

Caspase-3/7 activity measurement was performed as previously described (Yuste et al., 2001). Briefly, *TERT*-disrupted (*TERT* KO) or non-targeting NALM-6 cells were collected at different days in culture and lysed in NP-40 buffer [50 mM Tris-HCl pH 6.8, 150 mM NaCl, 1% (v/v) NP-40, 1X cOmplete EDTA-free protease inhibitor cocktail] and protein quantification was performed using the Lowry method before storage at -20 °C. On the day of the assay, lysates were thawed on ice and 25 µg of protein lysate was mixed on ice with 2X Caspase assay buffer [40 mM HEPES-NaOH pH 7.2, 300 mM NaCl, 10 mM EDTA, 0.2% (w/v) CHAPS, 2% (v/v) NP-40, 20% (w/v) sucrose, 20 mM DTT, 50 µM Ac-DEVD-AFC]. Assay plates were incubated at 35 °C for 20h and fluorescence intensity (excitation: 400 nm, emission: 505 nm) was measured every hour using a Tecan M1000pro plate reader. Relative caspase-3/7 activity was calculated as the slope of fluorescence intensity increase over time in the indicated samples relative to wild-type NALM-6 cells.

Telomeric Restriction Fragment length (TRF) analysis

Telomere length was assessed as previously described (Chu et al., 2016). Briefly, genomic DNA was extracted from NALM-6 cells and treated with Proteinase K (0.1 mg/mL) before digestion with *Hinf*I and *Rsa*I. The electrophoresis of the digested DNA was performed in a 0.7% (w/v)

agarose gel in 0.5X TBE (Tris/Borate/EDTA) for 16 h at 70V before gel denaturation, neutralization and drying. The hybridization was performed with [γ-^{32}P] ATP-labeled $(C_3TA_2)_3$ probe followed by washing and autoradiography on a phosphor storage screen and scanned using a Typhoon FLA-9500 phosphorimager (GE healthcare). The signal intensity in each line was measured using FIJI and the mean TRF length for each sample was calculated using the formula $(\Sigma(OD_i) / \Sigma(OD_i/L_i))$, where OD is the lane intensity at position *i* and L is the DNA size at position *i* as extrapolated from the *Hin*dIII-digested Lambda DNA ladder.

Competitive growth assays

Competitive growth assays of *TERT*-disrupted (*TERT* KO) cells were performed as described previously by transducing cells with LentiCRISPRv2-GFP lentiviral particles targeting *TERT* at a low multiplicity of infection (MOI) to obtain a transduction efficiency between 30-70% (Benslimane et al., 2020). Cells were propagated by sub-culturing every 3 days and were monitored for the percentage of *TERT* KO cells by flow cytometry on a BD FACSCanto II after FSC/SSC gating and indel sequencing. The ratio (R_d) of *TERT* KO cells (N_{KO}) to wild-type cells (N_{WT}) at a given day (d) is described by the following formula:

$$R_d = \frac{N_{KO} \times 2^{d \times G_{KO}}}{N_{WT} \times 2^{d \times G_{WT}}}$$

This formula is equivalent to:

$$R_d = R_0 \times 2^{\,d(G_{KO} - G_{WT})}$$

The ratio R_t was log-transformed (base 2) and linear regression relative to days in culture was used to extract the slope. This slope was used to calculate the growth rate of *TERT* KO cells for

each genotype G_{KO} and normalized to the growth rate of wild-type cells (G_{WT} = 1.25 doublings/day on average for NALM-6 cells) to obtain the relative fitness value (F):

$$F = \frac{G_{KO}}{G_{WT}}$$

Two-population competitive growth assays were used to measure the relative cell fitness of *TERT* KO NALM-6 cells, either as a single knockout in an otherwise wild-type background, or in the presence of a second, separately disrupted gene (see below). For relative fitness measurement of cells lacking *TERT* alone, or cells lacking *TERT* and another gene (B), the relative fitness is described by the following equation:

$$R_{d;B} = R_{0;B} \times 2^{\,d(G_{KO;B} - G_B)}$$

Where $G_{KO;B}$ describes the growth rate of cells in which *TERT* disruption is induced via CRISPR transduction as described above in NALM-6 cells already containing a disruption of another gene (*B*), and G_B is the number of doublings per day of NALM-6 cells lacking only gene *B*. In this context, relative fitness is given by the ratio of the growth rate of the *TERT* KO cell population in background B ($G_{KO;B}$) to the growth rate of the same *TERT* KO population in a wild-type NALM-6 background ($G_{KO;WT}$):

$$F_B = \frac{G_{KO;B}}{G_{KO;WT}}$$

To investigate how chemical perturbations affect the relative fitness of *TERT* KO NALM-6, cells were seeded at 1 x 10^5 cells/mL and treated with the respective compounds for 6 days (sub-cultured 3 days after compound addition with fresh compound) and EGFP-positive cells percentage was measured (at day 0, 3 and 6 of compound treatment) on a BD FACSCanto II after

FSC/SSC gating. Similar to the modelling described above, the effect of chemical perturbations on relative fitness can be modeled using the two following equations:

$$R_{d;cmpd} = R_0 \times 2^{\,d(G_{KO;cmpd} - G_{WT;cmpd})}$$

$$R_{d;DMSO} = R_0 \times 2^{\,d(G_{KO;DMSO} - G_{WT;DMSO})}$$

Where $G_{WT;DMSO}$ equals 1.25 doublings/day for NALM-6 cells and $G_{WT;cmpd}$ is the number of doublings that WT cells undergo in the compound treatment (derived from the average relative proliferation of WT cells transduced with non-targeting sgRNAs). $G_{KO;DMSO}$ is the growth rate of *TERT* KO cells without any chemical perturbation and $G_{KO;cmpd}$ is the value of interest and corresponds to the growth rate of *TERT* KO cells when treated with a given compound. Relative fitness is given by the following formula:

$$F_{cmpd} = \frac{G_{KO;cmpd} / G_{KO;DMSO}}{G_{WT;cmpd} / G_{WT;DMSO}}$$

Four-population competitive growth assay:

Four-population competitive growth assays were performed as previously described with the following modifications (Horlbeck et al., 2018). NALM-6 cells were transduced simultaneously with LentiCRISPRv2-GFP and LentiCRISPRv2-mCherry lentiviral particles at a low multiplicity of infection (MOI) to obtain all 4 possible populations. Cells were propagated for 14 days to ensure indel formation approached a plateau and were subsequently seeded at 1 x 10^5 cells/mL and treated with the respective compounds for the indicated time (sub-cultured every 2-3 days with fresh compound) with measurement of the percentage of EGFP- and mCherry-positive cells by flow cytometry on a BD LSRFortessa analyzer after FSC/SSC gating. At each timepoint, $\log_2$-

transformed sgRNA enrichment was calculated by first taking the ratio of percentage of cells in each population (EGFP-positive, mCherry-positive, double-positive) to the percentage of WT cells (double-negative) and then normalizing by the ratio at day 0 before log-transformation of the normalized ratio (base 2).

Western blots

Immunoblotting was performed as previously described (Benslimane et al., 2020). Briefly, 30 µg of protein lysates were resolved on a 10% (v/v) SDS-PAGE gel and transferred to a 0.22 µm nitrocellulose membrane. Membranes were blocked with 3% (w/v) milk in TBS-Tween 0.05% (v/v) (TBST) before blotting using the following antibodies diluted in TBST: p53 (1:1000), C16orf72 (TAPR1; 1:2000), GAPDH (1:5000), α-Tubulin (1:5000) followed by incubation with the respective HRP-conjugated secondary antibodies (1:10000 dilution in 3% (w/v) milk in TBST). Blots were developed by incubation with ECL substrate and chemiluminescence was measured using a ChemiDoc MP (Bio-Rad). Specific antibodies used are listed under Key Resources.

qPCR measurement of mRNA relative expression

Cells were collected after the indicated treatments and resuspended in QIAzol and RNA extraction using the miRNeasy mini kit following the manufacturer's instructions. Reverse transcription was performed on 0.5 µg of RNA after a treatment with DNAse I to remove any remaining genomic DNA followed by reverse transcription with random hexamers (ThermoFisher, #SO142) using the SuperScript IV enzyme following the manufacturer's instructions and cDNA was diluted 1:15 in ddH_2O before storage at -20 °C. qPCR reactions were

carried out by adding 3 µL of the diluted cDNA to 5 µL of PowerUP SYBR 2X mastermix and 2 µL of primer mixes for each target transcript (2 µM of each primer) before incubation in the Viia7 (Applied Biosystems) using the following program: 2 min at 95 °C followed by 40 cycles of 3 sec at 95 °C and 30 sec at 60 °C. Relative expression was measured using the $\Delta\Delta C_T$ method by using both housekeeping genes as internal controls as previously described (Vandesompele et al., 2002). Please see Supplementary Methods for specific primer sequences (Cuella-Martin et al., 2016).

RNA-seq Read Analysis

Reads were aligned with Bowtie2.2.5 (with default parameters) to all RefSeq transcripts (as at April 2018). Alignments with fewer than 2 inserted or deleted bases and a maximum edit distance of 5 (i.e. the sum of inserted, deleted, or mismatched bases) were counted to generate read counts per transcript and reads mapping to transcripts produced from the same gene locus were summed together to generate read counts per gene. The tabulated read counts per gene in knockout cells were analyzed for differential expression relative to non-targeting controls using the DESeq2 pipeline with the SARtools package on R (Love et al., 2014; Varet et al., 2016). A random selection of 1000 non-differentially expressed genes was included in volcano plots to aid visualization.

Protein-protein interaction identification by BioID

The cDNA sequence of *C16ORF72* (*TAPR1*) was retrieved from the CCDS database (#10538.1), ordered as a gBlock dsDNA fragment from IDT and cloned into a 3XFLAG-miniTurbo plasmid

(provided by Brian Raught and Anne-Claude Gingras; a gift from Feng-Qian Li and Ken-Ichi Takemaru; Addgene #124647) under the control of a MNDU3 promoter (obtained from a modified version of pCCL-c-MNDU3-X, a gift from Donald Kohn; Addgene #81071) (Logan et al., 2004) using Gibson assembly (Branon et al., 2018; Gibson et al., 2009). Proximity labeling of TAPR1 interactors was carried out as described previously, with the following modifications (Meant et al., 2020). Briefly, NALM-6 expressing miniTurbo-TAPR1, miniTurbo-EGFP or miniTurbo-NLS-EGFP (3 replicates, 30 million cells per replicate) were grown in the presence of 50 µM biotin for 1h. Cells were washed to remove excess biotin and cells were lysed in RIPA buffer supplemented with Benzonase (250U), sonicated on ice and cleared by centrifugation before protein quantification using the Lowry method. 50 µL of pre-equilibrated Streptavidin-sepharose beads with RIPA buffer were incubated with 2 mg of protein lysate overnight at 4 °C with agitation followed with 3 washes with RIPA buffer to remove non-specific binding and 10 washes in PBS to remove any traces of detergent. Subsequent sample preparation and peptide identification were performed at the Institute for Research in Immunology and Cancer Proteomics Platform. Samples were reconstituted in 50 mM ammonium bicarbonate with 10 mM TCEP [Tris(2-carboxyethyl)phosphine hydrochloride; Thermo Fisher Scientific], and vortexed for 1 h at 37 °C. Chloroacetamide (Sigma-Aldrich) was added for alkylation to a final concentration of 55 mM. Samples were vortexed for another hour at 37 °C. One microgram of trypsin was added, and digestion was performed for 8 h at 37 °C. Samples were dried down and solubilized in 5% (v/v) acetonitrile (ACN)-0.2% (v/v) formic acid (FA). Peptides were loaded and separated on a reversed-phase column (150-µm i.d. by 200 mm) with a 56-min gradient from 10 to 30% (v/v) ACN-0.2% (v/v) FA and a 600-nl/min flow rate on an Easy nLC-1200 instrument connected

to a Q-Exactive HF Biopharma (Thermo Fisher Scientific, San Jose, CA). Each full MS spectrum acquired at a resolution of 60,000 was followed by tandem-MS (MS-MS) spectra acquisition on the most abundant multiply charged precursor ions for a maximum of 3s. Tandem-MS experiments were performed using higher-energy collisional dissociation (HCD) at a collision energy of 27%. The data were processed using PEAKS X (Bioinformatics Solutions, Waterloo, ON) and searched against a concatenated forward and reverse Uniprot human database (20349 entries). Mass tolerances on precursor and fragment ions were 10 ppm and 0.01 Da, respectively. Fixed modification was carbamidomethyl (C). Variable selected posttranslational modifications were oxidation (M), deamidation (NQ), phosphorylation (STY). The data were visualized with Scaffold 4.3.0 (protein threshold, 99%, with at least 2 peptides identified and a false-discovery rate [FDR] of 1% for peptides). Tabulated total peptide count for proteins identified in each sample were used for subsequent analysis after filtering to remove low-count proteins, with retention of proteins that were detected in 2 out of 3 replicates of a given bait and possessed a minimum of 5 peptides for a given bait. Median-ratio normalization was used to account for inter-sample total peptide count variability followed by log-transformation of the peptide counts (base 2, pseudo-count of 1 added to remove zeros) (Valikangas et al., 2018). Potential contaminants were filtered by removing proteins that are present in at least 20% of experiments reported in the CRAPome database V1.1 (as at May 30th 2020) (Mellacheruvu et al., 2013). The average log_2-transformed peptide counts for control baits (n=6) were subtracted from that of TAPR1 bait (n=3) to calculate the fold-change and the Welch t-test was used to calculate a p-value for each fold-change followed by p-value adjustment for multiple comparisons using the Benjamini-Hochberg

method to obtain an FDR. Proteins with an FDR below 0.1 and a fold-change higher than 2 were considered as potential TAPR1 interactors.

SUPPLEMENTARY KEY RESOURCES

REAGENT or RESOURCE	SOURCE	IDENTIFIER
**Antibodies**		
Rabbit anti-GAPDH [clone 14C10]	Cell Signalling Technologies	Cat# 2118; RRID: AB_561053
Rabbit anti-Alpha-Tubulin [clone EPR13478(B)]	Abcam	Cat# ab176560; RRID: AB_2860019
Goat anti-p53	Santa Cruz	Cat# sc-6243-G; RRID: AB_653753
Mouse anti-C16orf72 [clone 2B8]	ThermoFisher Scientific	Cat# TA501515; RRID: AB_11125795
Donkey anti-goat IgG-HRP	Santa Cruz	Cat# sc-2020; RRID: AB_631728
Goat anti-mouse IgG-HRP (H+L)	Promega	Cat# W4021; RRID: AB_430834
Goat anti-rabbit IgG-HRP (H+L)	Jackson immunoresearch	Cat# #111-035-003; RRID: AB_2313567
Bacterial and Virus Strains		
E. coli: Stbl3 strain	ThermoFisher	Cat#: C737303
**Chemicals, Peptides, and Recombinant Proteins**		
DMSO	Corning	Cat# 29-950-CQC
BIBR1532	SelleckChem, see also (Bashash et al., 2017; Damm et al., 2001; Liu et al., 2020; Nakashima et al., 2013; Pascolo et al., 2002)	Cat# S1186
NSC-687852	Cayman Chemical	Cat# 11324
WP-1130	Cayman Chemical	Cat# 15227
Atovaquone	Tocris Bioscience	Cat# 6358
Brequinar	AdooQ Biosciences	Cat# A12442-5
NBMPR	Cayman Chemical	Cat# 16403

Nucleosides	Millipore-Sigma	Cat# ES-008-D
Nutlin-3a	Sigma	Cat# SML0580
Doxorubicin	MedChem Express	Cat# HY-15142
Doxycycline	Sigma	Cat# D9891
PEI 25000	Polysciences Inc.	Cat# 23966-1
Protamine sulfate	Sigma	Cat# P4020
CellTiter-Glo	Promega	Cat# G7573
Benzamidine hydrochloride hydrate	Sigma	Cat# B6506
FastStart SYBR Green 2X mastermix	Roche	Cat# #4673484001
PowerUP SYBR 2X mastermix	ThermoFisher Scientific	Cat# A25776
cOmplete EDTA-free protease inhibitor cocktail	Sigma	Cat# 11873580001
Ac-DEVD-AFC	Cayman Chemical	Cat# 14459
ECL western blot substrate	ThermoFisher Scientific	Cat# 32109
FastDigest Esp3I	ThermoFisher Scientific	Cat# FD0454
FastAP	Fermentas	Cat# EF0651
KAPA HiFi HotStart enzyme	KAPA Biosystems	Cat# KK2502
Taq DNA ligase	Enzymatics	Cat# L6060F
Phusion High-Fidelity DNA Polymerase	Fisher Scientific	Cat# F530S
T5 exonuclease	NEB	Cat# M0363S
DpnI	NEB	Cat# R0176S
HinfI	ThermoFisher Scientific	Cat# IVGN071-6
RsaI	ThermoFisher Scientific	Cat# IVGN042-6
SuperScript IV reverse-transcriptase	ThermoFisher Scientific	Cat# 18090050
Streptavidin-sepharose beads	GE Healthcare	Cat# 17511301
Benzonase nuclease	Sigma	Cat# E1014-5KU
Trypsin	Promega	Cat# V511A
**Deposited Data**		

CRISPR knockout screen sgRNA sequences, RNA-seq sequence data, mass spectrometry raw data	This study	HTS data: https://www.ncbi.nlm.nih.gov/geo/query/acc.cgi?acc=GSE160869. MS data: https://www.ebi.ac.uk/pride, using identifiers PXD022128 and 10.6019/PXD022128
**Experimental Models: Cell Lines**		
NALM-6	(Han et al., 1979). Provided by Steve Elledge; genotype confirmed by authors, see also	RRID: CVCL_0092 https://web.expasy.org/cellosaurus/CVCL_0092
HEK293T	ATCC	Cat# CRL-3216; RRID: CVCL_0063
**Oligonucleotides**		
EKO library amplification PCR1 Forward: 5'-AGCGCTAGCTAATGCCAACTT-3'	(Bertomeu et al., 2017)	N/A
EKO library amplification PCR1 Reverse: 5'-GCCGGCTCGAGTGTACAAAA-3'	(Bertomeu et al., 2017)	N/A
EKO library amplification PCR2 – TruSeq Universal adapter -2: 5'-AATGATACGGCGACCACCGAGATCTACACTCTTTCCCTACACGACGCTCTTCCGATCTTGTGGAAAGGACGAAACA-3'	(Bertomeu et al., 2017)	N/A
EKO library amplification PCR2 – TruSeq Universal adapter 0: 5'-AATGATACGGCGACCACCGAGATCTACACTCTTTCCCTACACGACGCTCTTCCGATCTCTTGTGGAAAGGACGAAACA-3'	(Bertomeu et al., 2017)	N/A

EKO library amplification PCR2 – TruSeq Universal adapter +2: 5’-AATGATACGGCGACCACCGAGATCTACACTCTTTCCCTACACGACGCTCTTCCGATCTGACTTGTGGAAAGGACGAAACA-3’	(Bertomeu et al., 2017)	N/A
EKO library amplification PCR2 – TruSeq Universal adapter +5: 5’-AATGATACGGCGACCACCGAGATCTACACTCTTTCCCTACACGACGCTCTTCCGATCTCACACCTTGTGGAAAGGACGAAACA-3’	(Bertomeu et al., 2017)	N/A
EKO library amplification PCR2 – TruSeq Universal adapter +7: 5’-AATGATACGGCGACCACCGAGATCTACACTCTTTCCCTACACGACGCTCTTCCGATCTCTACAGACTTGTGGAAAGGACGAAACA-3’	(Bertomeu et al., 2017)	N/A
EKO library amplification PCR2 – TruSeq adapter with index: 5’-CAAGCAGAAGACGGCATACGAGAT(6bp index)GTGACTGGAGTTCAGACGTGTGC TCTTCCGATCCACCGACTCGGTGCCACTTTT-3’	(Bertomeu et al., 2017)	N/A
qTRAP ACX primer: 5’-GCGCGGCTTACCCTTACCCTTACCCTAACC-3'	(Herbert et al., 2006)	N/A
qTRAP TS primer: 5’-AATCCGTCGAGCAGAGTT-3'	(Herbert et al., 2006)	N/A
CDKN1A qPCR forward primer: 5’-CCTCATCCCGTGTTCTCCTTT -3’	(Cuella-Martin et al., 2016)	N/A
CDKN1A qPCR reverse primer: 5’-GTACCACCCAGCGGACAAGT -3’	(Cuella-Martin et al., 2016)	N/A
MDM2 qPCR forward primer: 5’-GGCCTGCTTTACATGTGCAA-3’	(Cuella-Martin et al., 2016)	N/A
MDM2 qPCR reverse primer: 5’-GCACAATCATTTGAATTGGTTGTC -3’	(Cuella-Martin et al., 2016)	N/A
BAX qPCR forward primer: 5’-CCTTTTCTACTTTGCCAGCAAAC-3’	(Cuella-Martin et al., 2016)	N/A
BAX qPCR reverse primer: 5’-GAGGCCGTCCCAACCAC-3’	(Cuella-Martin et al., 2016)	N/A
GAPDH qPCR forward primer: 5’-CAGCAACAGGGTGGTGGAC -3’	This study	N/A

GAPDH qPCR reverse primer: 5'-CATTGCTGGGGCTGGTG -3'	This study	N/A
HPRT1 qPCR forward primer: 5'-TGACACTGGCAAAACAATGCA -3'	This study	N/A
HPRT1 qPCR reverse primer: 5'-GGTCCTTTTCACCAGCAAGCT -3'	This study	N/A
TKOv3 library PCR 1 LCV2 forward: 5'-GAGGGCCTATTTCCCATGATTC-3'	(Hart et al., 2017)	N/A
TKOv3 library PCR 1 LCV2 forward: 5'-GTTGCGAAAAAGAACGTTCACGG-3'	(Hart et al., 2017)	N/A
TERT-targeting sgRNA #1: 5'-GCTGCGCAGCCACTACCGCG-3'	This study	N/A
TERT-targeting sgRNA #2: 5'-ACGAAGCCGTACACCTGCCA-3'	This study	N/A
TERT-targeting sgRNA #3: 5'-CCAAGAAGTTCATCTCCCTG-3'	This study	N/A
TAPR1-targeting sgRNA #1: 5'-GCCGTGGCCCAGCTCTACAA-3'	This study	N/A
TAPR1-targeting sgRNA #2: 5'-TTTGTAGAGATTGGTGACGG-3'	This study	N/A
TP53-targeting sgRNA #3: 5'-GAGAGAATCTCCGCAAGAAAG-3'	This study	N/A
AAVS1-targeting control sgRNA: 5'-GGGGCCACTAGGGACAGGAT-3'	(Benslimane et al., 2020)	N/A
AzGreen-targeting control sgRNA: 5'-GGCCACAACTTCGTGATCGA-3'	(Benslimane et al., 2020)	N/A
TERT-targeting sgRNA #1 PCR forward primer: 5'-CTTCACGTCCGGCATTCGT-3'	This study	N/A
TERT-targeting sgRNA #1 PCR reverse primer: 5'-AGGAAGAGGGGGTTCTCGTC-3'	This study	N/A
TERT-targeting sgRNA #1 sequencing primer: 5'-CTCCTTCAGGCAGGACAC-3'	This study	N/A
TERT-targeting sgRNA #2 PCR forward primer: 5'-CTTCACGTCCGGCATTCGT-3'	This study	N/A

TERT-targeting sgRNA #2 PCR reverse primer: 5'-AGGAAGAGGGGGTTCTCGTC-3'	This study	N/A
TERT-targeting sgRNA #2 sequencing primer: 5'-CGTGACGATGGAGACAGGAG-3,	This study	N/A
TERT-targeting sgRNA #3 PCR forward primer: 5'-CTTCACGTCCGGCATTCGT-3'	This study	N/A
TERT-targeting sgRNA #3 PCR reverse primer: 5'-AGGAAGAGGGGGTTCTCGTC-3'	This study	N/A
TERT-targeting sgRNA #3 sequencing primer: 5'-CGTGACGATGGAGACAGGAG-3'	This study	N/A
TAPR1-targeting sgRNA #1 PCR forward primer: 5'-GGCAGCGGTTATCTGGTCC-3'	This study	N/A
TAPR1-targeting sgRNA #1 PCR reverse primer: 5'-TAACATGCGCGCAGATGACT-3'	This study	N/A
TAPR1-targeting sgRNA #1 sequencing primer: 5'-GGCAGCGGTTATCTGGTCC-3'	This study	N/A
TAPR1-targeting sgRNA #2 PCR forward primer: 5'-GGCAGCGGTTATCTGGTCC-3'	This study	N/A
TAPR1-targeting sgRNA #2 PCR reverse primer: 5'-TAACATGCGCGCAGATGACT-3'	This study	N/A
TAPR1-targeting sgRNA #2 sequencing primer: 5'-AAAGTTCGTCCCACTTCCGA-3'	This study	N/A
AAVS1-targeting sgRNA #1 PCR forward primer: 5'-TGTGCCATCTCTCGTTTCTTA-3'	This study	N/A
AAVS1-targeting sgRNA #1 PCR reverse primer: 5'-CACAAAGGGAGTTTTCCACA-3'	This study	N/A
AAVS1-targeting sgRNA #1 sequencing primer: 5'-GTCATGGCATCTTCCAGGGGTC-3'	This study	N/A
**Recombinant DNA**		
LentiCRISPRv2GFP	Laboratory of David Feldser; (Walter et al., 2017)	Addgene, Cat# 82416; RRID: Addgene_82416
LentiCRISPRv2-mCherry	Laboratory of Agata Smorgorzewska	Addgene, Cat# 99154; RRID: Addgene_99154

LentiCRISPRv2-Puro	Laboratory of Feng Zhang; (Sanjana et al., 2014)	Addgene, Cat# 52961; RRID: Addgene_52961
psPAX2	Laboratory of Dider Trono	Addgene, Cat# 12260; RRID: Addgene_12260
pCMV-VSV-G	Laboratory of Bob Weinberg; (Stewart et al., 2003)	Addgene, Cat# 8454; RRID: Addgene_8454
C16orf72 (TAPR1) cDNA gBlock	IDT	N/A
pCCL-c-MNDU3-X	Laboratory of Donald Kohn, modified from (Logan et al., 2004)	Addgene, Cat# 81071; RRID: Addgene_81071
Flag-miniTurbo	Provided by B. Raught and A.C. Gingras; from Feng-Qian Li and Ken-Ichi Takemaru	Addgene, Cat# 124647; RRID: Addgene_124647
Flag-miniTurbo-TAPR1	This study	N/A
TKOv3 sgRNA library	Laboratory of Jason Moffat; (Hart et al., 2017)	N/A
**Software and Algorithms**		
sgRNA Designer	(Doench et al., 2016)	https://portals.broadinstitute.org/gpp/public/analysis-tools/sgrna-design
RANKS	(Bertomeu et al., 2017)	https://github.com/JCHuntington/RANKS
DrugZ	(Colic et al., 2019)	https://github.com/hart-lab/drugz
g:Profiler web server	(Raudvere et al., 2019)	https://biit.cs.ut.ee/gprofiler/gost
ICE (v2)	Synthego, (Brinkman et al., 2014) (for underlying algorithm)	https://ice.synthego.com/

Proteomics Identifications Database (PRIDE)	(Perez-Riverol et al., 2019)	https://www.ebi.ac.uk/pride/
Proteome Xchange Consortium	(Deutsch et al., 2017)	http://www.proteomexchange.org/
Prism	Graphpad	v8.0.2
R statistical software	Open source	https://www.r-project.org/; v3.4.0
Adobe Illustrator CC 2017	Adobe	v21.1.0
Bowtie	(Langmead & Salzberg, 2012)	http://bowtie-bio.sourceforge.net/index.shtml; v2.2.5
REVIGO	(Supek et al., 2011)	http://revigo.irb.hr/
PEAKS X	Bioinformatics Solutions	https://www.bioinfor.com/peaks-studio/
Scaffold	Proteome Software	V4.3.0; http://www.proteomesoftware.com/products/scaffold/
**Other**		
Fetal bovine serum	Wisent	Cat# 080150
SF Cell Line 4D-Nucleofector X Kit L	Lonza	Cat# V4XC-2012
PrepGEM Tissue kit	Zygem	Cat# PTI0200
DC protein assay	Bio-Rad	Cat# 5000112
Gentra Puregene Cell kit	Qiagen	Cat# 158388
miRNeasy mini kit	Qiagen	Cat# 217004
QIAzol	Qiagen	Cat# 79306
KAPA mRNAseq Hyperprep kit	KAPA Biosystems	Cat# KK8581
KAPA library quantification kit	KAPA Biosystems	Cat# KK4973

SUPPORTING INFORMATION REFERENCES

Bashash, D., Zareii, M., Safaroghli-Azar, A., Omrani, M. D., & Ghaffari, S. H. (2017). Inhibition of telomerase using BIBR1532 enhances doxorubicin-induced apoptosis in pre-B acute lymphoblastic leukemia cells. *Hematology, 22*(6), 330-340. doi:10.1080/10245332.2016.1275426

Benslimane, Y., Bertomeu, T., Coulombe-Huntington, J., McQuaid, M., Sanchez-Osuna, M., Papadopoli, D., Avizonis, D., Russo, M. S. T., Huard, C., Topisirovic, I., Wurtele, H., Tyers, M., & Harrington, L. (2020). Genome-Wide Screens Reveal that Resveratrol Induces Replicative Stress in Human Cells. *Mol Cell*. doi:10.1016/j.molcel.2020.07.010

Bertomeu, T., Coulombe-Huntington, J., Chatr-Aryamontri, A., Bourdages, K. G., Coyaud, E., Raught, B., Xia, Y., & Tyers, M. (2017). A High-Resolution Genome-Wide CRISPR/Cas9 Viability Screen Reveals Structural Features and Contextual Diversity of the Human Cell-Essential Proteome. *Mol Cell Biol, 38*(1). doi:10.1128/MCB.00302-17

Branon, T. C., Bosch, J. A., Sanchez, A. D., Udeshi, N. D., Svinkina, T., Carr, S. A., . . . Ting, A. Y. (2018). Efficient proximity labeling in living cells and organisms with TurboID. *Nat Biotechnol, 36*(9), 880-887. doi:10.1038/nbt.4201

Brinkman, E. K., Chen, T., Amendola, M., & van Steensel, B. (2014). Easy quantitative assessment of genome editing by sequence trace decomposition. *Nucleic Acids Res, 42*(22), e168. doi:10.1093/nar/gku936

Chu, T. W., D'Souza, Y., & Autexier, C. (2016). The Insertion in Fingers Domain in Human Telomerase Can Mediate Enzyme Processivity and Telomerase Recruitment to Telomeres in a TPP1-Dependent Manner. *Mol Cell Biol, 36*(1), 210-222. doi:10.1128/MCB.00746-15

Colic, M., Wang, G., Zimmermann, M., Mascall, K., McLaughlin, M., Bertolet, L., Lenoir, W. F., Moffat, J., Angers, S., Durocher, D., & Hart, T. (2019). Identifying chemogenetic interactions from CRISPR screens with drugZ. *Genome Med, 11*(1), 52. doi:10.1186/s13073-019-0665-3

Cuella-Martin, R., Oliveira, C., Lockstone, H. E., Snellenberg, S., Grolmusova, N., & Chapman, J. R. (2016). 53BP1 Integrates DNA Repair and p53-Dependent Cell Fate Decisions via Distinct Mechanisms. *Mol Cell, 64*(1), 51-64. doi:10.1016/j.molcel.2016.08.002

Damm, K., Hemmann, U., Garin-Chesa, P., Hauel, N., Kauffmann, I., Priepke, H., Niestroj, C., Daiber, C., Enenkel, B., Guilliard, B., Lauritsch, I., Muller, E., Pascolo, E., Sauter, G., Pantic, M., Martens, U. M., Wenz, C., Lingner, J., Kraut, N., Rettig, W. J., & Schnapp, A. (2001). A highly selective telomerase inhibitor limiting human cancer cell proliferation. *EMBO J, 20*(24), 6958-6968. doi:10.1093/emboj/20.24.6958

Deutsch, E. W., Csordas, A., Sun, Z., Jarnuczak, A., Perez-Riverol, Y., Ternent, T., Campbell, D. S., Bernal-Llinares, M., Okuda, S., Kawano, S., Moritz, R. L., Carver, J. J., Wang, M., Ishihama, Y., Bandeira, N., Hermjakob, H., & Vizcaino, J. A. (2017). The ProteomeXchange consortium in 2017: supporting the cultural change in proteomics public data deposition. *Nucleic Acids Res, 45*(D1), D1100-D1106. doi:10.1093/nar/gkw936

Doench, J. G., Fusi, N., Sullender, M., Hegde, M., Vaimberg, E. W., Donovan, K. F., Smith, I., Tothova, Z., Wilen, C., Orchard, R., Virgin, H. W., Listgarten, J., & Root, D. E. (2016).

Optimized sgRNA design to maximize activity and minimize off-target effects of CRISPR-Cas9. *Nat Biotechnol, 34*(2), 184-191. doi:10.1038/nbt.3437

Gibson, D. G., Young, L., Chuang, R. Y., Venter, J. C., Hutchison, C. A., 3rd, & Smith, H. O. (2009). Enzymatic assembly of DNA molecules up to several hundred kilobases. *Nat Methods, 6*(5), 343-345. doi:10.1038/nmeth.1318

Han, T., Dadey, B., & Minowada, J. (1979). Cultured human leukemic non-T/non-B lymphoblasts and their stimulating capacity in "one-way" mixed lymphocyte reaction: suggestive evidence for early T-cell or B-cell precursors. *Cancer, 44*(1), 136-140. doi:10.1002/1097-0142(197907)44:1<136::aid-cncr2820440124>3.0.co;2-i

Hart, T., Tong, A. H. Y., Chan, K., Van Leeuwen, J., Seetharaman, A., Aregger, M., Chandrashekhar, M., Hustedt, N., Seth, S., Noonan, A., Habsid, A., Sizova, O., Nedyalkova, L., Climie, R., Tworzyanski, L., Lawson, K., Sartori, M. A., Alibeh, S., Tieu, D., Masud, S., Mero, P., Weiss, A., Brown, K. R., Usaj, M., Billmann, M., Rahman, M., Constanzo, M., Myers, C. L., Andrews, B. J., Boone, C., Durocher, D., & Moffat, J. (2017). Evaluation and Design of Genome-Wide CRISPR/SpCas9 Knockout Screens. *G3 (Bethesda), 7*(8), 2719-2727. doi:10.1534/g3.117.041277

Herbert, B. S., Hochreiter, A. E., Wright, W. E., & Shay, J. W. (2006). Nonradioactive detection of telomerase activity using the telomeric repeat amplification protocol. *Nat Protoc, 1*(3), 1583-1590. doi:10.1038/nprot.2006.239

Horlbeck, M. A., Xu, A., Wang, M., Bennett, N. K., Park, C. Y., Bogdanoff, D., . . . Gilbert, L. A. (2018). Mapping the Genetic Landscape of Human Cells. *Cell, 174*(4), 953-967 e922. doi:10.1016/j.cell.2018.06.010

Langmead, B., & Salzberg, S. L. (2012). Fast gapped-read alignment with Bowtie 2. *Nat Methods, 9*(4), 357-359. doi:10.1038/nmeth.1923

Liu, C., Zhou, H., Sheng, X. B., Liu, X. H., & Chen, F. H. (2020). Design, synthesis and SARs of novel telomerase inhibitors based on BIBR1532. *Bioorg Chem, 102*, 104077. doi:10.1016/j.bioorg.2020.104077

Logan, A. C., Nightingale, S. J., Haas, D. L., Cho, G. J., Pepper, K. A., & Kohn, D. B. (2004). Factors influencing the titer and infectivity of lentiviral vectors. *Hum Gene Ther, 15*(10), 976-988. doi:10.1089/hum.2004.15.976

Love, M. I., Huber, W., & Anders, S. (2014). Moderated estimation of fold change and dispersion for RNA-seq data with DESeq2. *Genome Biol, 15*(12), 550. doi:10.1186/s13059-014-0550-8

Meant, A., Gao, B., Lavoie, G., Nourreddine, S., Jung, F., Aubert, L., . . . Roux, P. P. (2020). Proteomic Analysis Reveals a Role for RSK in p120-catenin Phosphorylation and Melanoma Cell-Cell Adhesion. *Mol Cell Proteomics, 19*(1), 50-64. doi:10.1074/mcp.RA119.001811

Mellacheruvu, D., Wright, Z., Couzens, A. L., Lambert, J. P., St-Denis, N. A., Li, T., . . . Nesvizhskii, A. I. (2013). The CRAPome: a contaminant repository for affinity purification-mass spectrometry data. *Nat Methods, 10*(8), 730-736. doi:10.1038/nmeth.2557

Nakashima, M., Nandakumar, J., Sullivan, K. D., Espinosa, J. M., & Cech, T. R. (2013). Inhibition of telomerase recruitment and cancer cell death. *J Biol Chem, 288*(46), 33171-33180. doi:10.1074/jbc.M113.518175

Pascolo, E., Wenz, C., Lingner, J., Hauel, N., Priepke, H., Kauffmann, I., Garin-Chesa, P., Rettig,

W. J., Damm, K., & Schnapp, A. (2002). Mechanism of human telomerase inhibition by BIBR1532, a synthetic, non-nucleosidic drug candidate. *J Biol Chem, 277*(18), 15566-15572. doi:10.1074/jbc.M201266200

Perez-Riverol, Y., Csordas, A., Bai, J., Bernal-Llinares, M., Hewapathirana, S., Kundu, D. J., Inuganti, A., Griss, J., Mayer, G., Eisenacher, M., Perez, E., Uszkoreit, J., Pfeuffer, J., Sachsenberg, T., Yilmaz, S., Tiwary, S., Cox, J., Audain, E., Walzer, M., Jarnuczak, A. F., Ternent, T., Brazma, A., & Vizcaino, J. A. (2019). The PRIDE database and related tools and resources in 2019: improving support for quantification data. *Nucleic Acids Res, 47*(D1), D442-D450. doi:10.1093/nar/gky1106

Raudvere, U., Kolberg, L., Kuzmin, I., Arak, T., Adler, P., Peterson, H., & Vilo, J. (2019). g:Profiler: a web server for functional enrichment analysis and conversions of gene lists (2019 update). *Nucleic Acids Res, 47*(W1), W191-W198. doi:10.1093/nar/gkz369

Sanjana, N. E., Shalem, O., & Zhang, F. (2014). Improved vectors and genome-wide libraries for CRISPR screening. *Nat Methods, 11*(8), 783-784. doi:10.1038/nmeth.3047

Stewart, S. A., Dykxhoorn, D. M., Palliser, D., Mizuno, H., Yu, E. Y., An, D. S., Sabatini, D. M., Chen, I. S., Hahn, W. C., Sharp, P. A., Weinberg, R. A., & Novina, C. D. (2003). Lentivirus-delivered stable gene silencing by RNAi in primary cells. *RNA, 9*(4), 493-501. doi:10.1261/rna.2192803

Supek, F., Bosnjak, M., Skunca, N., & Smuc, T. (2011). REVIGO summarizes and visualizes long lists of gene ontology terms. *PLoS One, 6*(7), e21800. doi:10.1371/journal.pone.0021800

Valikangas, T., Suomi, T., & Elo, L. L. (2018). A systematic evaluation of normalization methods in quantitative label-free proteomics. *Brief Bioinform, 19*(1), 1-11. doi:10.1093/bib/bbw095

Vandesompele, J., De Preter, K., Pattyn, F., Poppe, B., Van Roy, N., De Paepe, A., & Speleman, F. (2002). Accurate normalization of real-time quantitative RT-PCR data by geometric averaging of multiple internal control genes. *Genome Biol, 3*(7), RESEARCH0034. doi:10.1186/gb-2002-3-7-research0034

Varet, H., Brillet-Gueguen, L., Coppee, J. Y., & Dillies, M. A. (2016). SARTools: A DESeq2- and EdgeR-Based R Pipeline for Comprehensive Differential Analysis of RNA-Seq Data. *PLoS One, 11*(6), e0157022. doi:10.1371/journal.pone.0157022

Walter, D. M., Venancio, O. S., Buza, E. L., Tobias, J. W., Deshpande, C., Gudiel, A. A., . . . Feldser, D. M. (2017). Systematic In Vivo Inactivation of Chromatin-Regulating Enzymes Identifies Setd2 as a Potent Tumor Suppressor in Lung Adenocarcinoma. *Cancer Res, 77*(7), 1719-1729. doi:10.1158/0008-5472.CAN-16-2159

Yuste, V. J., Bayascas, J. R., Llecha, N., Sanchez-Lopez, I., Boix, J., & Comella, J. X. (2001). The absence of oligonucleosomal DNA fragmentation during apoptosis of IMR-5 neuroblastoma cells: disappearance of the caspase-activated DNase. *J Biol Chem, 276*(25), 22323-22331. doi:10.1074/jbc.M100072200
